# Novel T cell/organoid culture system allows *ex vivo* modeling of intestinal graft-versus-host disease

**DOI:** 10.3389/fimmu.2023.1253514

**Published:** 2023-08-29

**Authors:** Diana M. Matthe, Martin Dinkel, Benjamin Schmid, Tina Vogler, Markus F. Neurath, Hendrik Poeck, Clemens Neufert, Maike Büttner-Herold, Kai Hildner

**Affiliations:** ^1^ Department of Medicine 1, Kussmaul Campus for Medical Research, University Hospital Erlangen, University of Erlangen-Nuremberg, Erlangen, Germany; ^2^ Deutsches Zentrum Immuntherapie (DZI), University Hospital Erlangen, Erlangen, Germany; ^3^ Optical Imaging Centre Erlangen (OICE), University of Erlangen-Nuremberg, Erlangen, Germany; ^4^ Clinic and Polyclinic for Internal Medicine III, University Hospital Regensburg, Regensburg, Germany; ^5^ Department of Nephropathology, Institute of Pathology, Friedrich-Alexander-University Erlangen-Nuremberg (FAU) and University Hospital, Erlangen, Germany

**Keywords:** graft-versus-host disease, GvHD, allogeneic hematopoietic stem cell transplantation, intestinal organoids, epithelial cell death, alloreactive T cell, *ex vivo* model, intraepithelial lymphocytes

## Abstract

Acute graft-versus-host disease (GvHD) remains the biggest clinical challenge and prognosis-determining complication after allogeneic hematopoietic stem cell transplantation (allo-HSCT). Donor T cells are acceptedly key mediators of alloreactivity against host tissues and here especially the gut. In support of previous studies, we found that the intestinal intra-epithelial lymphocyte (IEL) compartment was dynamically regulated in the course of MHC class I full mismatch allo-HSCT. However, while intestinal epithelial cell (IEC) damage endangers the integrity of the intestinal barrier and is a core signature of intestinal GvHD, the question whether and to what degree IELs are contributing to IEC dysregulation is poorly understood. To study lymphoepithelial interaction, we employed a novel *ex vivo* T cell/organoid co-culture model system. Here, allogeneic intra-epithelial T cells were superior in inducing IEC death compared to syngeneic IEL and allogeneic non-IEL T cells. The ability to induce IEC death was predominately confined to TCRβ+ T cells and was executed in a largely IFNγ-dependent manner. Alloreactivity required a diverse T cell receptor (TCR) repertoire since IELs genetically modified to express a TCR restricted to a single, non-endogenous antigen failed to mediate IEC pathology. Interestingly, minor histocompatibility antigen (miHA) mismatch was sufficient to elicit IEL-driven IEC damage. Finally, advanced live cell imaging analyses uncovered that alloreactive IELs patrolled smaller areas within intestinal organoids compared to syngeneic controls, indicating their unique migratory properties within allogeneic IECs. Together, we provide here experimental evidence for the utility of a co-culture system to model the cellular and molecular characteristics of the crosstalk between IELs and IEC in an allogeneic setting *ex vivo*. In the light of the emerging concept of dysregulated immune-epithelial homeostasis as a core aspect of intestinal GvHD, this approach represents a novel experimental system to e.g. screen therapeutic strategies for their potential to normalize T cell/IEC- interaction. Hence, analyses in pre-clinical *in vivo* allo-HSCT model systems may be restricted to hereby positively selected, promising approaches.

## 1 Introduction

GvHD (graft-versus-host diesase) is a common and severe complication after hematopoietic stem cell transplantation (HSCT) which affects up to 50% of patients and represents the primary cause of post-transplant mortality (1, 2). Pathomechanistically, donor T lymphocytes are considered to function as the central effector cell mediating GvHD development and progression. These so-called alloreactive T cells are activated by recognizing foreign (allogeneic) host antigens presented on MHC molecules, which leads to their secretion of inflammatory cytokines, amongst others IL-2 or IFNγ (3). In turn, these cytokines can induce tissue damage either directly, but also indirectly by sensitizing or activating other immune cells and thereby amplifying the inflammatory process (4).

Recent progress in the field has revealed that T cells and T cell-derived mediators are not only driving systemic and local inflammatory circuits but also actively drive GvHD-associated epithelial cell damage in the gut (5, 6). This is critical as, apart from the skin, the gastrointestinal (GI) tract is most frequently affected, and involvement of the lower GI tract is most associated with non-relapse mortality (7). GI GvHD may clinically present with nausea, diarrhea, abdominal cramps or gastrointestinal bleeding, causing considerable morbidity (8). Moreover, the T cell-mediated destruction of the intestinal epithelium abrogates critical mucosal barrier functions, thereby exacerbating patients’ burden by causing malabsorption and malnutrition (8).

Mechanistically, especially T cell-derived type II interferon, IFNγ, has been identified to directly interfere with intestinal epithelial cell homeostasis as it negatively regulates intestinal stem cell maintenance and recovery in the course of intestinal GvHD (5). Interestingly, despite the fact that the intra-epithelial lymphocyte (IEL) compartment contains a significant IFNγ-expressing T cell pool naturally populating the intestinal epithelial cell (IEC) layer (9), the cell type-specific contribution of IELs in the context of IFNγ-driven epithelial cell pathology has not been studied in detail.

Small intestinal (SI) IELs represent a heterogenous cell pool that largely consists of T lymphocytes, which are assumed to be predominately tissue-resident, antigen-experienced T cells (10–12). They can be subdivided into induced (CD4^+^ or CD8αβ^+^ TCRαβ^+^) and natural (CD4^-^CD8αβ^-^ or CD8αα^+^, TCRαβ^+^ or TCRγδ^+^) IEL subsets (10, 13). While the development of natural IELs is not yet fully understood, they are considered to predominantly respond to self-antigens rather than foreign ligands (10, 13). Induced IELs originate from conventional CD4^+^ or CD8αβ^+^ TCRαβ^+^ MHC-restricted T cells that underwent selection in the thymus and migrated to the gut after encountering non-self antigens in the periphery (10, 13). Due to their unique positioning at the mucosal barrier site, IELs are functionally tissue site-specific memory cells involved in the local protection against recurring infections, thereby ensuring the integrity of the intestinal epithelial lining. However, IELs have been also implicated to exert harmful pro-inflammatory effects under pathologic conditions as e.g. in inflammatory bowel disease or coeliac disease (9, 10, 13).

In the context of allo-HSCT, previous studies by others found that donor T cells indeed populate the recipient IEL compartment (14–16). However, the nature and characteristics of IEL/IEC interaction in the small intestine in the allogeneic setting have not been addressed yet. This is in part attributed to technical difficulties, since IELs are prone to apoptosis *ex vivo* (17). However, groundbreaking work showing that small intestinal (SI) organoids closely mimicking small intestinal epithelial cell structures found *in vivo* can be generated and expanded *ex vivo* from Lgr5^+^ intestinal stem cells (18) has paved the way for novel methodological approaches to study IEL biology. So progress by our group and others (17, 19, 20) demonstrating that SI organoids offer an appropriate microenvironment to stably culture IELs *ex vivo* has opened up new avenues to analyze IEL biology under physiological conditions, i.e. within the intestinal epithelial layer.

Here we sought to further apply this experimental concept to establish and initially characterize the SI IEL/IEC cross-talk in the allogeneic setting *ex vivo*. Data obtained by employing this novel allogeneic IEL/IEC co-culture model system demonstrate unique functional and migratory properties of IEL-resident T cells and disclose their significant impact on IEC integrity. We propose this model system to be a robust experimental platform providing so far undisclosed insight into IEL/IEC interaction potentially leading to the discovery of new therapeutic targets for the treatment of GvHD in the future.

## 2 Materials and methods

### 2.1 Mice

Mouse lines used in this study were either purchased from commercial vendors (Charles River Laboratories, Janvier Labs, The Jackson Laboratory) or bred in-house. The animals were kept in individually ventilated cages under specific-pathogen free conditions in the animal facilities of either Präklinisches Experimentelles Tierzentrum (PETZ) or Department of Medicine 1, Universitätsklinikum Erlangen. C.B10-H2 b/LilMcdJ mice and B6.129S7-Ifngtm1Ts/J mice were purchased from The Jackson Laboratory. CD45.1/Ly5.1 B6.SJL-*Ptrprc^a^Pepc^b^*/BoyCrl and C57BL/6-Tg(TcraTcrb)1100Mjb/Crl (OTItg) mice were obtained from Charles River Laboratories. C57BL/6J mice are referred to as “B6” throughout the study. Experiments using T cells isolated from Ifng^+/+^ and Ifng^+/-^ littermates yielded comparable results. Therefore, data from mice of both genotypes were pooled and used as a “Ifng^+/+^” control group for Ifng^-/-^ mice. This study was carried out in accordance with the current legislation and the guidelines of the government of Lower and Middle Franconia and animal experiments were approved by the government of Middle Franconia in Bavaria, Germany (54.2532.1 – 24/11-3).

### 2.2 Murine allo-HSCT and induction of acute GvHD *in vivo*

GvHD was induced as described before (21, 22). In short, female 10-12 week old Balb/c mice were lethally irradiated with 8 Gy total body irradiation at d0. On the next day, recipient mice received 5 × 10^6^ T cell depleted bone marrow (BM) cells from CD45.1/Ly5.1 B6.SJL-*Ptprca Pepcb*/BoyCrl mice intravenously (i.v.). On d2 after irradiation, recipient mice were i.v. injected with 0.7 × 10^6^ allogeneic CD3^+^ splenocytes from B6 wildtype mice. BM and splenocyte cell suspensions were enriched for the desired cell populations using magnetic separation and anti-CD90.2 microbeads (Miltenyi Biotec) or the Mouse Pan T Cell Isolation Kit II (Miltenyi Biotec), respectively, according to the manufacturer’s instructions. Control animals only received T cell depleted BM, but no CD3^+^ splenocytes.

### 2.3 Isolation of intestinal crypts and organoid culture

To generate organoids, small intestinal (SI) crypts were isolated from female B6 or Balb/c mice (>8 weeks) to obtain stem cells for the establishment of organoid cultures. For this, mice were sacrificed, the small intestine was removed and placed in cold PBS. The intestine was then flushed with cold PBS, opened longitudinally and the villi were carefully scratched off using a cover slip. The organ was then cut into 0.5 cm pieces and put into a tube with cold PBS. The tissue pieces were thoroughly washed by pipetting up and down with a 25 ml serological pipette, repeating this step with fresh PBS until the solution was clear. SI tissue was then digested for 30-40 min at 4 °C on a MACSmix Tube Rotator (Miltenyi Biotec) in 30 ml PBS + 2 mM EDTA. After the incubation time, the digestion solution was removed by passing through a 100 μm cell strainer. The remaining tissue pieces were then placed into 10 ml fresh cold PBS and vigorously vortexed to mechanically isolate the intestinal crypts. Crypts were collected on ice by passing through a 70 μm cell strainer. The tissue pieces were mixed again with 10 ml fresh cold PBS and the previous step was repeated for a total of five times. The intestinal stem cell-containing crypts were then pelleted by centrifugation at 300 g, 4°C for 5 min and washed one time with PBS and one time with *basal crypt media* (BCM) consisting of Advanced DMEM/F-12 (Gibco) + 1% Penicillin/Streptomycin (Sigma-Aldrich) + 10 mM HEPES (Sigma-Aldrich) + 1% GlutaMAX (Gibco). The presence of successfully isolated crypts was confirmed in a cell culture microscope (Leica DMIL LED) followed by plating crypts in a 1:1 mixture of BCM with Matrigel (Corning) in 24-well-plates. After polymerization of the Matrigel dome at 37° C for 25-30 min, 0.5 ml *crypt culture media* (CCM) consisting of BCM + 1X B-27 supplement (Gibco) + 1 mM N-Acetyl-L-cysteine (Sigma-Aldrich) + 20 ng/ml rmEGF (Immunotools) + 100 ng/ml rmNoggin (PeproTech) + 10% R-Spondin (culture supernatant from R-Spondin producing cell line) was added per well.

To study cytokine-mediated effects on Fas expression regulation by organoids in isolation, organoids generated as described above were passaged by washing in cold PBS and then resuspended in 50 μl of a 1:1 mixture of PBS and Matrigel (Corning). After plating the organoids in a 48-well-plate and polymerization of the Matrigel dome, 300 μl of CCM containing either no additional cytokines as unstimulated control, 10 ng/ml rmTNFα (Immunotools), 10 ng/ml rmIFNγ (Peprotech) or a combination of 10 ng/ml rmTNFα + 10 ng/ml rmIFNγ was added per well. On d2, organoids were harvested and single cell suspensions of organoids were generated as described below (cf. 2.6.4) and stained with EpCAM-Alexa Fluor 488 (Biolegend) and Fas-APC (Biolegend) for 15 min at 4°C in FACS buffer (PBS + 3% FCS).

### 2.4 Isolation of T cells

T cells for co-cultures were isolated from naïve, unmanipulated mice. As IEL or splenocyte donors, mice irrespective of their sex and between 8 and 17 weeks (mean: 11.2 weeks) of age were used.

#### 2.4.1 Isolation and magnetic-activated cell sorting (MACS)-mediated enrichment of SI IEL T cells

For the isolation of SI IELs, mice were sacrificed and the SI was taken and kept in PBS solution on ice. The intestine was flushed with cold PBS and Peyer’s patches were removed. The organ was opened longitudinally, washed by vigorously moving it in PBS, and then cut into 0.5 cm pieces which were placed into 20 ml of pre-warmed predigestion solution consisting of HBSS (Sigma-Aldrich) + 10 mM HEPES + 5 mM EDTA + 5% FCS (Pan Biotech) + freshly added 1 mM Dithiothreitol (Sigma-Aldrich). After 20 min incubation at 37 °C on a shaker, the predigestion solution containing the tissue pieces was vortexed for 20-30 sec and then passed through a 100 μm cell strainer. The flow-through fraction containing IELs was collected and kept on ice. The tissue pieces were placed again in 20 ml of fresh predigestion solution and the described step was repeated one more time. Next, the remaining tissue pieces were incubated for 20 min at 37 °C on a shaker in 10 ml of HBSS + 10 mM HEPES. In the meantime, approximately 35 ml of the collected flow-through from the predigestion steps were transferred into a new tube, thereby discarding any debris that had sedimented on the bottom. After the last incubation step, the tissue pieces were vortexed and passed through a 100 μm cell strainer. Next, leukocytes were isolated by density centrifugation. For this, the pooled flow-through from the predigestion steps and the HBSS steps was pelleted by centrifugation at 300 g, 4 °C, 5 min, resuspended in 40 % Percoll (Cytiva) in complete media (DMEM high glucose (Gibco) + 10 % FCS + 1 % Penicillin/Streptomycin) and overlayed on 70 % Percoll in HBSS. After centrifugation at 2500 rpm, RT, 20 min without brakes, lymphocytes enriched in the interphase were collected and washed in MACS buffer (PBS + 2 mM EDTA + 0.5 % bovine serum albumin (Sigma-Aldrich)).

To further enrich for T cells of interest within the IEL fraction, positive magnetic cell separation was performed. For this, IELs were labeled either with anti-CD3-Biotin (Biolegend), anti-CD3-PE (Biolegend), anti-CD8a-PE (Biolegend), anti-CD8a-Biotin (Biolegend), anti-TCRγδ-PE (Biolegend) or anti-TCRβ-PE (Biolegend) for 15 min at 4 °C depending on the application as indicated in the figure legends. The cells were then washed with 2 ml MACS buffer, resuspended in 80 μl MACS buffer and 20 μl of respective magnetic beads (anti-PE MicroBeads UltraPure (Miltenyi Biotec) or anti-Biotin MicroBeads (Miltenyi Biotec)) per 10^6^ cells and incubated for 15 min at 4 °C. After another washing step with 2 ml MACS buffer, the cells were resuspended in 500 μl MACS buffer and passed through pre-wetted magnetic LS columns, which were washed with 3 x 3 ml MACS buffer. The flow-through fraction containing unlabeled cells was discarded, whereas the magnetically labeled cells of interest were collected by flushing the column with a plunger upon removal of the column from the magnet. Purity was checked by flow cytometry and was routinely >95 % of CD3^+^ cells within lymphocytes. After magnetic separation, T cells were resuspended in BCM for further steps.

#### 2.4.2 Isolation of splenocytes

Splenocytes were obtained by removing the spleen from sacrificed mice, followed by dissociating the splenic tissue by mashing and passing it through a 40 μm cell strainer. After centrifugation, red cell lysis was performed by resuspending the cell pellet in 3 ml ammonium-chloride-potassium lysis buffer (0.15 M NH_4_Cl, 10 mM KHCO_3,_ 100 μM Na_2_EDTA) for 3 min. To optimize purity while also maintaining comparability in treatment between IELs and splenocytes, T cells were first enriched by negative magnetic separation using the Pan T Cell Isolation Kit II, mouse (Miltenyi Biotec) according to the manufacturer’s protocol and in a second step, positive magnetic cell enrichment (anti-CD8a-Biotin, Biolegend) as described above (2.4.1) was performed.

### 2.5 Allogeneic T cell/organoid co-culture

Allogeneic co-cultures were started 2-4 days after crypt isolation. At this time point, grown organoids were harvested from the 24-well-plates with cold PBS, washed twice with cold PBS and resuspended in BCM. Approximately 100 organoids were mixed with 2.5 x 10^5^ magnetically enriched T cells (cf. 2.4.1/.2) in BCM in a 48-well-plate and incubated for 30 min at 37 °C. After that, organoids and T cells were harvested again from the well with cold PBS, spun down, resuspended in 50 μl of a 1:1 mixture of PBS and Matrigel (Corning) and plated in a 48-well-plate. Lastly, after polymerization of the Matrigel dome, 300 μl of CCM + 10 ng/ml rmIL-7 (Immunotools) + 10 ng/ml rmIL-15 (Immunotools or R&D systems) + 100 IU/ml recombinant human (rh) IL-2 (Immunotools) were added per well.

### 2.6 Analyses after co-culture

Endpoint analyses were routinely performed on day 2 after start of the co-culture, unless indicated otherwise.

#### 2.6.1 Fluorometric cell death quantification

Fluorometric quantification of cell death among organoids after co-culture was based on Bode, Mueller et al., 2019 (23). In more detail, cell-free supernatants were harvested by carefully pipetting off the liquid phase. The organoids were stained for 30 min at 37 °C in 250 μl/well BCM + 10 μg/ml propidium iodide (Invitrogen) + 10 μg/ml Hoechst 33342 (Invitrogen). After incubation, the wells were carefully washed three times with warm PBS to avoid disruption of the Matrigel dome. Fluorescence intensities within the wells were measured in PBS using a Tecan infinite M200 platereader in the setting “multiple reads per well (circle (filled))” with 1 mm border. For propidium iodide (PI) detection, the excitation wavelength was set to 535 nm and the emission wavelength 617 nm. Hoechst dye was excited at 361 nm and its emission was detected at 486 nm. After the platereader measurement, PBS was replaced by CCM and fluorescence microscopy pictures were taken on a Leica DMI4000 B inverted microscope. The intensity signal of the cell-permeable dye Hoechst was used to normalize for cell numbers in each well, while the extent of the uptake of the cell-impermeable dye PI reported the amount of cell death per well. Thereby, the PI/Hoechst ratio reports the relative cell death present in an assayed well, importantly in a – due to this normalization algorithm – seeding density-independent manner. To correct for physiological baseline cell death, the measured basal cell death, i.e. PI/Hoechst ratio from the respective organoid controls cultured without T cells was subtracted from PI/Hoechst ratios obtained by fluorometric microplate reader measurement from wells of the syngeneic (B6 organoids) and allogeneic (Balb/c organoids) co-culture conditions.

#### 2.6.2 Digital image analysis

To quantify the PI/Hoechst ratios in microscopic images, three representative pictures were taken on a Leica DMI4000 B inverted microscope with a 5X objective and analyzed in Fiji/ImageJ software (version 2.1.0/1.5.3v) by splitting the color channels and measuring the mean intensities for each of the fluorescent dyes. Mean intensities of PI were then normalized to Hoechst intensity, as described above (2.6.1).

#### 2.6.3 Three-dimensional (3D)-rendered images

To obtain 3D-rendered images of co-cultures, B6 (allogeneic) CD3+ enriched IELs were stained with 1 μM Cell Proliferation Dye eFluor 670 (eBioscience) according to the protocol provided by the manufacturer on d0 prior co-culturing. Then, co-cultures of labeled T cells and previously grown B6 (syngeneic)- or Balb/c (allogeneic)-derived SI organoids were started by plating in a µ-Slide 4 Well chamber slide (ibidi). On d2, co-cultures were stained with PI and Hoechst and washed as described above (2.6.1.). Z-stacks of whole organoids were taken at a Leica TCS SP5 II confocal microscope with a 63X objective, with 1 μm distance between slices.

Post-processing of imaging data was performed in Fiji/ImageJ software (version 2.1.0/1.5.3v). To improve the resolution in z, the number of slices was interpolated to approximately match the voxel depth to the pixel width and pixel height. 3D-rendering and -animation were achieved using the plugin 3Dscript (24).

#### 2.6.4 Flow cytometry

To prepare single-cell suspensions for flow cytometry, co-cultures were washed with cold PBS and incubated for 3 x 5 min at 37 °C in 1 ml TrypLE (Gibco). In between incubation steps, co-cultures were vortexed vigorously for 10 sec to mechanically dissociate the organoids. After the last incubation step, TrypLE was stopped by addition of cold PBS, and the cell suspension was spun down (300 g, 5 min, 4 °C). Prior to staining, cells were strained through a 100 μm mesh to remove any residual cell aggregates. To assess apoptotic cells after co-culture, cells were first incubated with CD3-APCCy7 (Biolegend), EpCAM-Alexa Fluor 488 (Biolegend) and Fas-APC (Biolegend) for 15 min at 4 °C in FACS buffer (PBS + 3 % FCS) and then washed with FACS buffer. Then, they were stained for 15 min at RT with Annexin V in Binding Buffer from the eBioscience™ Annexin V Apoptosis Detection Kit eFluor™ 450 (Invitrogen). For the analysis of IEL subpopulations and FasL expression after co-culture, cells were stained with TCRβ-Pacific Blue (Biolegend), TCRγδ-PECy7 (Biolegend), CD4-Brilliant Violet 605 (Biolegend, 100451), CD8a-APCCy7 (Biolegend), CD8b-Alexa Fluor 700 (Biolegend) for 15 min at 4 °C in FACS buffer.

To measure granzyme B and perforin protein expression in T cells by flow cytometry, an intracellular staining protocol was applied. Briefly, single cell suspensions resuspended in DMEM (gibco) + 1 % P/S + 10 % FCS were plated in a 48-well-plate and stimulated with 50 ng/ml phorbol myristate acetate (PMA, Sigma-Aldrich) and 1 μM ionomycin (Sigma-Aldrich) at 37 °C. After 1 h, 1 mg/ml Brefeldin A (Sigma) was added to the cell culture followed by additional 3 h incubation at 37 °C. Then, cells were harvested, washed and stained with a LIVE/DEAD™ Fixable Aqua Dead Cell Stain Kit (Invitrogen) according to the manufacturer’s protocol. Next, cells were stained extracellularly with TCRβ-Pacific Blue (Biolegend) and TCRγδ-PECy7 (Biolegend) for 15 min at 4 °C in FACS buffer. After that, cells were fixed in 2 % formaldehyde (Carl Roth) in PBS for 15 min at RT. Subsequent to fixation, cells were washed with FACS buffer and resuspended in 0.05 % saponin in FACS buffer for permeabilization. Cells were stained intracellularly with Perforin-APC (Biolegend) and Granzyme B-FITC (Biolegend) in 0.5 % saponin in FACS buffer for 30 min at 4 °C followed by extensive washing steps prior resuspension in FACS buffer and analysis at the FACS machine.

Stained cells were measured on a LSRFortessa™ Cell Analyzer (BD) and analyzed in FlowJo™ software (FlowJo™ Software (for Windows) [software application] Version 10.6.2. Ashland, OR: Becton, Dickinson and Company; 2019).

#### 2.6.5 Real-time quantitative PCR

For gene expression analysis after co-culture, supernatants were removed and organoids were harvested using 350 μl RLT lysis buffer (Qiagen) + 4 % Dithiotreitol (Sigma-Aldrich) per well. RNA was isolated using the RNeasy Micro Kit (Qiagen) and measured on a Nanodrop. Then, 500-1000 ng of RNA were reversely transcribed to cDNA using the iScript™ cDNA Synthesis Kit (Bio-Rad). Realtime-PCR was performed using iQ™ SYBR^®^ Green Supermix (Bio-Rad) in a CFX Connect or CFX96 Real-Time PCR Detection system (Bio-Rad). Primers included: *Hprt* (for: TGGATACAGGCCAGACTTTGTT, rev: CAGATTCAACTTGCGCTCATC) *Lgr5* (for: GACTTTAACTGGAGCAAAGATCTCA; rev: CGAGTAGGTTGTAAGACAAATCTAGC), *Olfm4* (Mm_Olfm4_2_SG QuantiTect Primer Assay Qiagen, QT01557052), *Ifng* (for: ATCTGGAGGAACTGGCAAAA, rev: TGAGCTCATTGAATGCTTGG), *Tnf* (for: CTTGTGGCAGGGGCCACCAC, rev: CCATGCCGTTGGCCAGGAGG), *Gzmb* (Mm_Gzmb_1_SG QuantiTect Primer Assay Qiagen, QT00114590), *Prf1* (for: CCACTCCAAGGTAGCCAAT, rev: GGAGATGAGCCTGTGGTAAG), *Fasl* (for: CGTGAGTTCACCAACCAAAG, rev: TGTGTCTTCCCATTCCAGAG). Data were analyzed by employing the ddCT method. For epithelial-specific genes (*Lgr5, Olfm4*), the expression levels in the co-cultures were normalized so that the expression levels detected in the respective wells of organoids cultured without T cells equaled 1. That means, the syngeneic condition (B6 T cells + B6 organoids) was normalized to the expression level of B6 organoids alone and the allogeneic sample (B6 T cells + Balb/c organoids) was normalized to the Balb/c organoid w/o T cell control. For the T cell-centered gene expression analyses (*Ifng, Tnf, Gzmb, Prf1, Fasl*), gene expression levels were normalized to levels detected in samples derived from syngeneic co-culture.

#### 2.6.6 Enzyme-linked immunosorbent assay

Cell-free supernatants were assayed for IFNγ protein concentrations *via* ELISA using the IFN gamma Mouse Uncoated ELISA Kit (Invitrogen) according to the manufacturer’s protocol and measured in a Tecan infinite M200 platereader. For the quantification of murine IL-2 protein concentration in cell-free supernatants, the ELISA MAX™ Standard Set Mouse IL-2 (Biolegend) was used according to the manufacturer’s protocol and measured in a Tecan infinite M200 platereader. It was experimentally verified that this kit was specifically detecting murine IL-2 but not recombinant human IL-2 that was added as a media supplement (cf. 2.5).

#### 2.6.7 Live imaging and migration analysis

Live imaging of the co-culture was performed on d1 on a Zeiss Spinning Disc Axio Observer Z1 microscope at the Optical Imaging Centre Erlangen (OICE) as described before (25). In short, B6 (allogeneic) CD3+ IELs were stained with 1 μM Cell Proliferation Dye eFluor 670 (eBioscience) according to the manufacturer’s protocol on d0 and co-cultures of labeled IELs and previously grown B6 (syngeneic)- or Balb/c (allogeneic)-derived SI organoids were plated in a µ-Slide 4 Well chamber slide (ibidi). During the imaging process, the samples were incubated in a 37 °C, 5 % CO_2_ chamber and pictures were taken every 30 sec for 45 min using a 25X objective.

Subsequent analysis was also performed as described before (19): Data was imported into Fiji/ImageJ software (version 2.1.0/1.5.3v) using Bio-Formats (26). To assess T cell migration within organoids, the plugin TrackMate (27) was applied with the following settings: LoG (Laplacian of Gaussian) detector with an estimated blob diameter of 7 μm and a threshold of 40-120 (depending on experiment), Simple LAP tracker (Linear Assignment Problem) with linking maximum distance and gap-closing maximum distance both set to 15 μm and gap-closing maximum frame gap of 2 μm. For speed analysis, tracks with a speed below 0.05 μm per second were excluded. To calculate the organoid area patrolled by T cells within the recorded time frame of 45 min, an in-house Fiji macro was applied to the output of TrackMate to sum up the area enclosed by 7 μm around the T cell tracks and relating it to the epithelial area as determined by a manually drawn region of interest, omitting the organoid lumen.

#### 2.6.8 Immunohistochemistry

Staining for CD3ε protein expression was performed on paraffin-embedded co-cultures or SI tissue sections from mice previously undergoing allo-HSCT as indicated (cf. section 2.2). For this purpose, co-cultures were washed with cold PBS, resuspended in HistoGel™ Specimen Processing Gel (Epredia), fixed in ROTI^®^Histofix 4.5 % formaldehyde (Carl Roth) and embedded in paraffin. After deparaffinization, slides were pre-treated by cooking the slides 2.5 min in Target Retrieval Solution pH 6 (Dako). After blocking endogenous peroxidase by incubation with 30 % H_2_O_2_ (Carl Roth) for 10 min and subsequently blocking with Avidin/Biotin for 15 min each (Avidin/Biotin Blocking Kit, Vector Laboratories), the slides were incubated with the primary antibody (rat anti-CD3, Bio-Rad) diluted 1:200 in 1% BSA overnight. Thereafter, slides were washed and incubated with the secondary antibody (biotinylated rabbit anti-rat IgG, Vector Laboratories) diluted 1:500 for 30 min. For signal detection, slides were treated with the VECTASTAIN^®^ Elite^®^ ABC-HRP Kit, Peroxidase (Vector Laboratories) according to the manufacturer’s protocol and with the ImmPACT^®^ DAB Substrate Kit, Peroxidase (HRP) (Vector Laboratories) as chromogen for 2.5 min. Finally, slides were counterstained with hematoxylin. Representative images were taken on a Leica DMI4000 B inverted microscope.

#### 2.6.9 Immunofluorescence

For immunofluorescence staining of cleaved caspase-3 protein, co-cultures were plated in µ-Slide 8 Well chamber slides (ibidi) on d0. Cleaved caspase-3 staining was performed on d1. For this, samples were fixed for 30-40 min with 4 % paraformaldehyde in PBS at RT, washed with PBS, permeabilized for 10 min with 0.1 % Triton X-100 (Sigma-Aldrich) in PBS at RT and blocked with histobuffer (10 % FCS, 5 % BSA in PBS) for 1 h at RT. Organoids were stained with anti-cleaved Caspase 3 AF648 (BD Biosciences) and anti-EpCAM-AF488 (BioLegend) diluted 1:100 and 1:200, respectively, in histobuffer overnight at 4 °C. The next day, the samples were washed three times with PBS. Nuclei were counterstained for 5 min with Hoechst 33342 (Invitrogen) diluted 1:10000 in PBS. After washing one time with A.d., the slides were mounted with Mowiol 4-88 (Carl Roth). Images were taken with a Leica TCS SP5 II confocal microscope using a 63X objective.

#### 2.6.10 Statistical analysis

Statistical analyses were performed using GraphPad Prism software (version 9.5.1). For the comparison of two groups, a two-tailed unpaired t test was employed with a significance level of 5 %. In case of statistical inequality of variances, a Welch’s correction was applied to the t test where indicated in the figure captions. For the comparison of more groups, a one-way ANOVA with a *post-hoc* analysis (Šídák’s multiple comparisons test) with a significance level of 5 % was applied.

## 3 Results

### 3.1 Allogeneic IEL T cells co-cultured with SI organoids *ex vivo* mirror *in vivo* behavior of donor T cells

**Figure 1:**
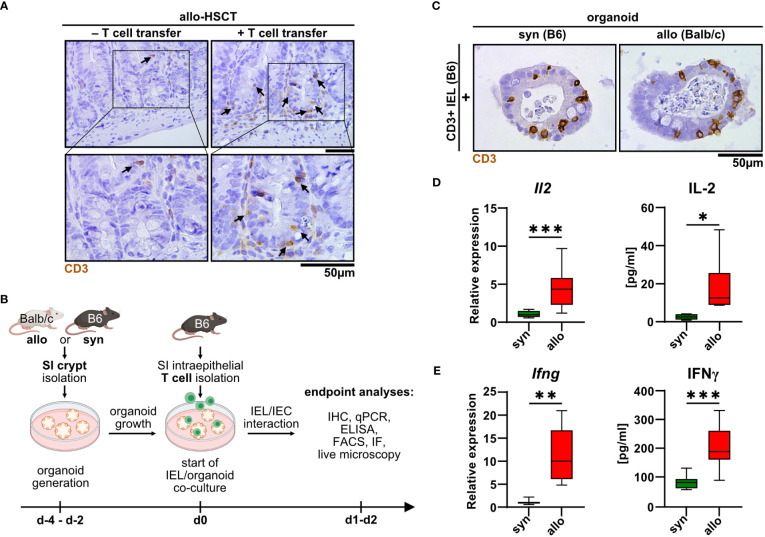
Upon allo-HSCT, small intestinal epithelium is infiltrated by allogeneic T cells, which can be mimicked in a novel IEL/organoid co-culture model system *ex vivo.* **(A)** Representative images show immunohistochemical stainings for CD3 protein from the small intestine (SI) of mice in an *in vivo* model of GvHD 30 days after lethal irradiation and rescue through transfer of T cell-depleted bone marrow cells from CD45.1/Ly5.1 B6.SJL-*Ptprc* ^a^ *Pepc* ^b^/BoyCrl mice. Mice additionally receiving allogeneic C57BL/6 CD3^+^ splenocytes (+ T cell transfer) serve as experimental group developing GvHD, while untreated mice represent controls without GvHD signs (- T cell transfer). Upper row contains inserts within the images indicating crypt areas that are displayed with magnification in the bottom row. **(B)** Schematic illustration of *ex vivo* IEL/organoid co-culture model workflow. SI organoids from Balb/c or C57BL/6 donor mice are generated from SI Lgr5^+^ stem cell containing crypts and grown for 2-4 days (d-4 – d-2). Allogeneic (Balb/c) organoids or syngeneic (C57BL/6) control organoids are then co-cultured with 2.5 x 10^5^ SI IEL T cell pools freshly isolated from C57Bl/6 donor mice (d0) or were left untreated as controls (not depicted here). Indicated endpoint analyses were performed 1 or 2 days after the start of the co-culture and included immunohistochemistry (IHC), quantitative PCR (qPCR), ELISA, flow cytometry (FACS), immunofluorescence (IF) stainings and live microscopy imaging. Created with BioRender.com. **(C)** Representative images showing immunohistochemical stainings of previously magnetically enriched SI CD3^+^ IELs of C57BL/6 mice (B6) within paraffin-fixed SI organoids on d2 after co-culturing as described in **(B)**, i.e. under allogeneic (Balb/c organoids) or syngeneic (B6 organoids) conditions are displayed. **(D, E)** IEL/organoid co-cultures were generated and cultured under syngeneic and allogeneic conditions as described in **(B)**. At d2, co-cultures were harvested, cell-free supernatant collected and total RNA was isolated from the IEL/organoid total cell pool. **(D)** *Il2* gene expression levels (left panel, n = 13) were determined by qPCR. Obtained data were normalized so that the relative amount detected within syngeneic culture equaled 1. Right panel displays IL-2 protein levels determined by ELISA within cell-free supernatants of syngeneic vs. allogeneic co-cultures (n = 7). **(E)** Left panel shows *Ifng* gene expression levels as determined by qPCR and normalized to the syngeneic condition (n = 8). Right panel depicts IFNγ protein levels (n = 9) in syngeneic vs. allogeneic co-cultures as measured by ELISA in cell-free supernatants. Graphs show median, minimum and maximum values in box and whiskers plots and represent pooled data from independent experiments, *p ≤ 0.05, **p ≤ 0.01, ***p ≤ 0.001 by two-tailed unpaired t test with Welch’s correction.

Intestinal T cells play a major role in the pathogenesis of intestinal GvHD. However, detailed insight into the disease-specific role of anatomically distinct T cell subsets is sparse. As previously shown by others (14–16), we confirmed that the SI epithelial layer of mice previously undergoing allo-HSCT using a MHC class I full mismatch model system is densely populated with T cells compared to control mice receiving T cell-depleted, allogeneic bone marrow alone (**Figure 1A**). While this correlative data suggests that IELs may contribute to the dysfunction of small intestinal epithelial cells (IECs) during intestinal GvHD, functional evidence in support of this assumption is lacking. To further explore and characterize allogeneic IEL/IEC interaction *ex vivo*, we adopted a previously reported syngeneic IEL/IEC co-culture model system (19). For this, we magnetically isolated T cells from the SI IEL fraction of healthy naïve B6 mice and cultured those within allogeneic SI organoids that were previously generated *ex vivo* from intestinal stem cell-containing small intestinal crypt preparations of Balb/c (allogeneic) or B6 mice (syngeneic controls) (**Figure 1B**). On day 2 of co-culture, both allogeneic and syngeneic organoids were densely populated by CD3^+^ IELs (**Figure 1C**). Organoid-residing allogeneic IELs expressed significantly higher levels of IL-2 and IFNγ compared to syngeneic T cells both on the transcriptional and on protein level (**Figures 1D, E**). Overall, our data indicate that IEL-derived allogeneic T cells can be cultured within SI organoids *ex vivo* and show signs of alloreactive activation.

### 3.2 Allogeneic IEL/IEC co-cultures are characterized by significantly increased cell death rates

**Figure 2:**
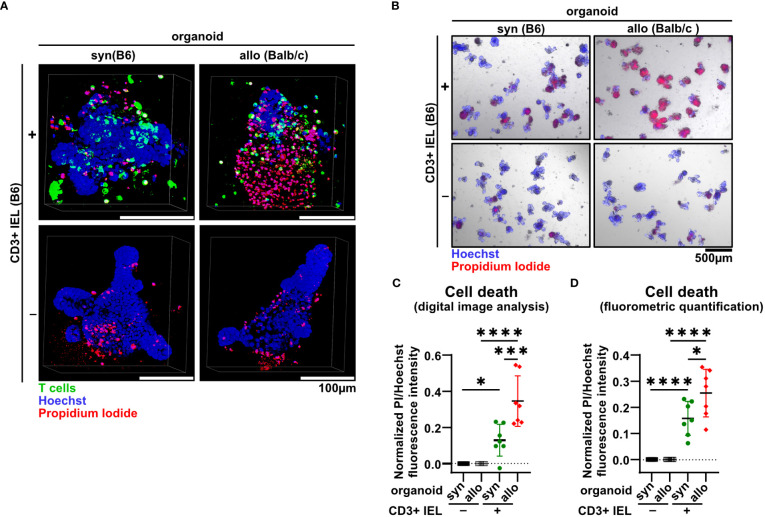
IELs induce significantly more organoid cell death under allogeneic compared to syngeneic co-culture conditions. **(A)** Representative 3D-reconstructed images of 2.5 x 10^5^ SI IELs from C57BL/6 (B6) mice enriched for CD3^+^ cells and then co-cultured (+) with syngeneic (B6) or allogeneic (Balb/c) organoids as well as control organoids, i.e. w/o T cells (-) on d2. Prior to co-culturing, T cells were fluorescently labeled with Proliferation Dye eFluor 670 (green). Organoids were stained with Hoechst dye (blue) to visualize cell nuclei while propidium iodide (red) was used to mark dead cells. Z-stacks were taken with a confocal Leica SP5 microscope and reconstructed using Fiji software and the 3Dscript plugin. **(B)** Representative examples of cell death assessment within co-cultures by combined staining with propidium iodide (red) and Hoechst (blue) on d2 of allogeneic vs syngeneic co-cultures or controls (w/o T cells) as described in **(A)** are depicted. Subsequent quantification of cell death rates within organoids was achieved by analysis of the fluorescence intensity in microscopic images using **(C)** Fiji software or **(D)** directly within an individual well using a microplate reader. In **(C, D)**, for normalization, the intensity of propidium iodide (PI) was related to the Hoechst signal (cf. methods section). To account for physiological, spontaneous cell death events, the mean PI/Hoechst ratios were normalized to the respective value obtained within organoid controls cultured in the absence of T cells. Graphs show the mean ± SD from n = 7 independent experiments. * p ≤ 0.05, *** p ≤ 0.001, **** p < 0.0001 by one-way ANOVA and Šídák’s multiple comparisons test.

The detection of cell death events especially of IECs represents one of the histopathological hallmarks of acute intestinal GvHD (5). Hence, we sought to establish a methodology allowing us to assay, visualize and reliably quantify cell death rates in the IEL/IEC co-culture model system. For this, at day 2, co-cultures were exposed to the dead cell-dye propidium iodide (PI) while we counterstained cell nuclei with Hoechst. As shown in **Figure 2A** and **Supplementary Video 1** by confocal microscopy scanning, organoids cultured alone, i.e. in the absence of T cells, contained only few and small areas with PI-positive cells presumably reflecting physiological cell debris shedding into the crypt lumina. Strikingly, the addition of fluorescently labeled allogeneic T cells led to a noticeable increase of PI^+^ cells. Importantly, PI^+^ areas were anatomically not restricted to the crypt lumen as it appeared to be the case for the majority of the events visible in syngeneic IEL/IEC co-cultures. This data suggested that the observed cell death in allogeneic IEL/IEC co-cultures exceeds physiological epithelial cell turnover as well as cell death due to occasional T cell activation in the syngeneic setting. To address that and objectively quantify cell death rates longitudinally across different experiments, we employed two related analysis protocols as described in detail in the method section. Starting from PI/Hoechst stained co-cultures (**Figure 2B**), we performed in parallel digital image analysis and fluorometry by normalization of fluorescent signals stemming from PI^+^ cells relative to the total cell pool per well represented by Hoechst fluorescence intensity. This fluorometric approach previously reported and validated by Bode, Mueller et al., 2019 (23) allows an objective quantification of organoid death, which, unlike e.g. assays based on metabolic changes due to cell death, is independent of the seeding density. As shown in **Figures 2C, D**, both assays uniformly revealed that an increase of cell death rates was already detectable after adding syngeneic IELs compared to organoids alone. However, allogeneic IELs induced cell death significantly more, exceeding the level observed after adding syngeneic T cells. Together, allo-IEL/IEC cultures are characterized by a significant increase of cell death events that can be reliably assessed and quantified by the application of PI/Hoechst staining assays.

### 3.3 Allogeneic CD3^+^ IEL-induced apoptosis of IECs largely accounts for enhanced cell death *ex vivo*

**Figure 3:**
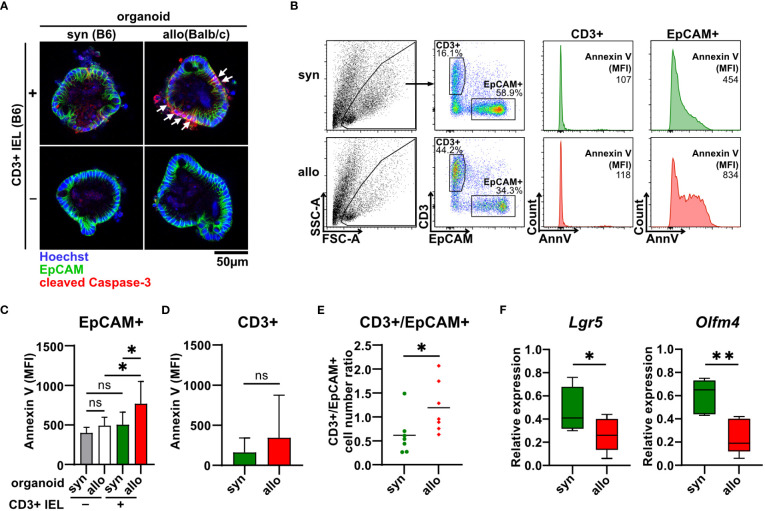
Allo-reactive IELs mediate apoptotic IEC death and dysregulation of the intestinal stem cell compartment *ex vivo*. **(A)** 2.5 x 10^5^ SI IELs enriched for CD3^+^ T cells from C57BL/6 (B6) mice were co-cultured (+) with syngeneic (B6) or allogeneic (Balb/c) organoids *ex vivo*. As controls, indicated organoids were cultured w/o T cells (-). To assess apoptotic cell death, co-cultures and controls were stained at d1 for cleaved caspase-3 (red) as well as EpCAM (green) and Hoechst (blue). Representative immunofluorescence stainings are displayed. **(B-E)** Single cell suspensions of co-cultures generated as described under **(A)** were analyzed at d2 of co-culture by flow cytometry. In **(B)**, the gating strategy of flow cytometric analysis of early apoptotic events within CD3^+^ T cells and EpCAM^+^ IECs by Annexin V staining is shown. Graph in **(C)** displays MFI for Annexin V staining of EpCAM^+^ cells; in **(D)** MFI for Annexin V staining within CD3^+^ cells is illustrated while in **(E)** cell count ratio of CD3^+^ and EpCAM^+^ cells is depicted. Graphs in **(C-E)** show mean ± SD from n = 7 biological replicates of independent experiments; **(C)**, *p ≤ 0.05 by one-way ANOVA and Šídák’s multiple comparisons test; **(D)** by two-tailed unpaired t test with Welch’s correction; **(E)**, *p ≤ 0.05 by two-tailed unpaired t test. **(F)** On d2, co-cultures executed as described under **(A)** were harvested and quantitative gene expression profiling of *Lgr5* (n = 8) and *Olfm4* (n = 5) was performed. For this, expression levels were normalized to levels detected within organoids cultured w/o T cells. Graphs show median, minimum and maximum in box and whiskers plots of pooled data of indicated, independent experiments, ns (not significant) p > 0.05, *p  ≤ 0.05, **p ≤ 0.01 by two-tailed unpaired t test.

To shed further light on the cellular compartment contributing to the elevated cell death rates, we stained organoids cultured with or without syn- or allogeneic IELs for the early apoptotic marker cleaved caspase 3. Interestingly, areas corresponding to the epithelial cell layer as identified by membrane-specific expression of the epithelial cell-specific molecule EpCAM (epithelial cell adhesion molecule) stained positive for cleaved caspase 3 (**Figure 3A**). We further performed flow cytometric analyses of single-cell suspensions of IELs (CD3+) and IEC (EpCAM+) after two days of allogeneic and syngeneic co-culture, respectively (**Figure 3B**). We found that the apoptotic IEC pool isolated from co-cultures with allogeneic but not syngeneic IELs showed significantly elevated Annexin V binding on their cell surface (**Figure 3C**). In contrast, the mean fluorescence intensity (MFI) of Annexin V bound to T cells was not increased in allogeneic vs. syngeneic settings (**Figure 3D**). This data strongly suggests that primarily IEC death accounts for the overall increased cell death fraction after two days of alloreactive IEL/organoid co-culture. Consistent with this interpretation, the T cell/IEC ratio significantly increased in allogeneic compared to syngeneic co-culture conditions (**Figure 3E**). In addition to our finding of increased IEC death, we wondered whether impaired intestinal stem cell homeostasis might contribute to this shift. Employing *Lgr5* and *Olfm4*, two molecular markers routinely used to robustly identify small intestinal stem cells (28, 29), we observed significantly reduced expression levels of both *Lgr5* and *Olfm4* upon co-culture with allogeneic IELs compared to syngeneic controls (**Figure 3F**). Overall, our data indicate that allogeneic IELs mediate IEC apoptosis and interfere with the integrity of intestinal epithelial stem cells resulting in a progressive loss of IECs within two days of co-culture.

### 3.4 Allo-T cell-derived IFNγ drives IEC death and regulates the intestinal epithelial stem cell niche

**Figure 4:**
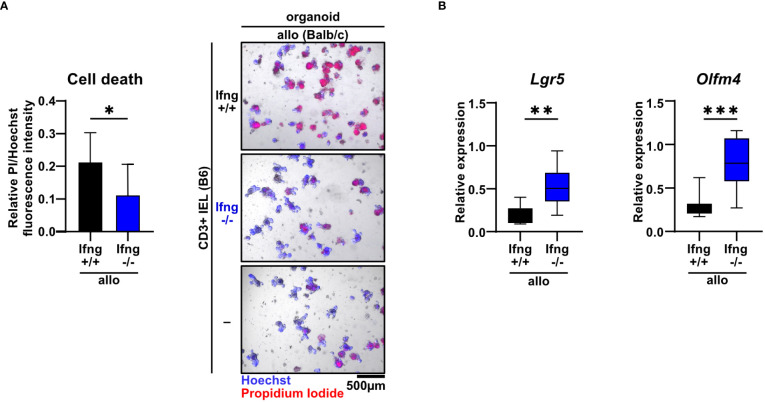
Dysregulation of the small intestinal epithelial cell and stem cell compartment in allogeneic co-cultures is dependent on IFNγ. **(A)** 2.5 x 10^5^ SI IELs enriched for CD3^+^ T cells from C57BL/6 (B6) IFNγ-sufficient or IFNγ-deficient mice were co-cultured with allogeneic (Balb/c) organoids *ex vivo*. As controls, Balb/c organoids were cultured alone, i.e. w/o T cells (-). At d2, fluorometric cell death quantification (left) was performed, with the PI/Hoechst fluorescence intensity ratio normalized to the wells with organoids cultured without T cells; representative microscopic images (right) of organoids double-stained with Hoechst and PI after co-culture are displayed. Graph depicts pooled data from n = 9 independent experiments, mean ± SD. *p  ≤ 0.05 by two-tailed unpaired t test. **(B)** IEL/IEL co-cultures were performed for 2d as described in **(A)**. Then, total cells were harvested and quantitative gene expression profiling of the stem cell markers *Lgr5* and *Olfm4* was performed. For this, expression levels were normalized to levels detected within organoids cultured w/o T cells. Pooled data from n = 8 independent experiment are depicted as median with minimum and maximum values in box and whiskers plots. **p ≤ 0.01, ***p ≤ 0.001 by two-tailed unpaired t test.

Type II interferon IFNγ is one of the key effector molecules of cytotoxic T cells but its contribution to intestinal GvHD-associated gut pathology remains controversial (30). Given our finding that allogeneic compared to syngeneic T cells express elevated IFNγ levels when co-cultured with IECs (**Figure 1D**), we sought to determine its functional impact on allo-IEL-mediated IEC death. Importantly, IFNγ-deficient IELs resulted in significantly less organoid death compared to IFNγ-proficient T cells (**Figure 4A**). Moreover, the intestinal epithelial stem cell compartment previously shown to be sensitive to IFNγ-mediated damage (31) was protected in co-culture with IFNγ-deficient compared to control allo-IELs as shown by significantly enhanced and hence largely recovered *Lgr5* and *Olfm4* expression levels (**Figure 4B**). Apart from IFNγ, T cells might additionally employ other soluble factor- or cell-contact-mediated mechanism to execute cytotoxic IEC death, as suggested by several lines of evidence in the literature (32). Assessing TNFα expression on RNA and protein level, we did not observe a differential regulation in our setting (**Supplementary Figure 1A**). In contrast, granzyme B/perforin gene expression levels were moderately, but significantly increased in allogeneic vs. syngeneic co-culture conditions (**Supplementary Figure 1B**), but we were unable to monitor equivalent changes in allogeneic IELs on the protein level (**Supplementary Figure 1C**). Finally, we evaluated whether the FasL/Fas (CD95L/CD95) axis may be involved in mediating T cell-induced target cell death in this model system. Similar to the data on cytotoxic molecules, FasL gene expression was significantly increased in allogeneic compared to syngeneic co-cultures, a finding that could not be confirmed on the protein level (**Supplementary Figure 2A**). In contrast, however, Fas receptor expression on EpCAM+ epithelial cells was significantly increased in co-cultures with allogeneic compared to syngeneic IELs (**Supplementary Figure 2B**). To assess whether IFNγ is able to regulate the pool of Fas+ IECs in this setting, we treated organoids with recombinant IFNγ. Strikingly, IFNγ treatment of organoids alone, i.e. in the absence of T cells, strongly increased the pool of Fas+ EpCAM+ epithelial cells (**Supplementary Figure 2C**). Overall, this data suggests that allogeneic IEL-derived IFNγ is a key molecular signal driving allogeneic IEC death. Mechanistically, IFNγ putatively increases the susceptibility of IECs by sensitizing IECs for FasL/Fas-mediated T cell-driven IEC death thereby strongly interfering with intestinal epithelial cell and stem cell homeostasis.

### 3.5 TCRαβ^+^CD8α^+^ IEL-derived T cells are the major executers of allo-mediated IEC death

**Figure 5:**
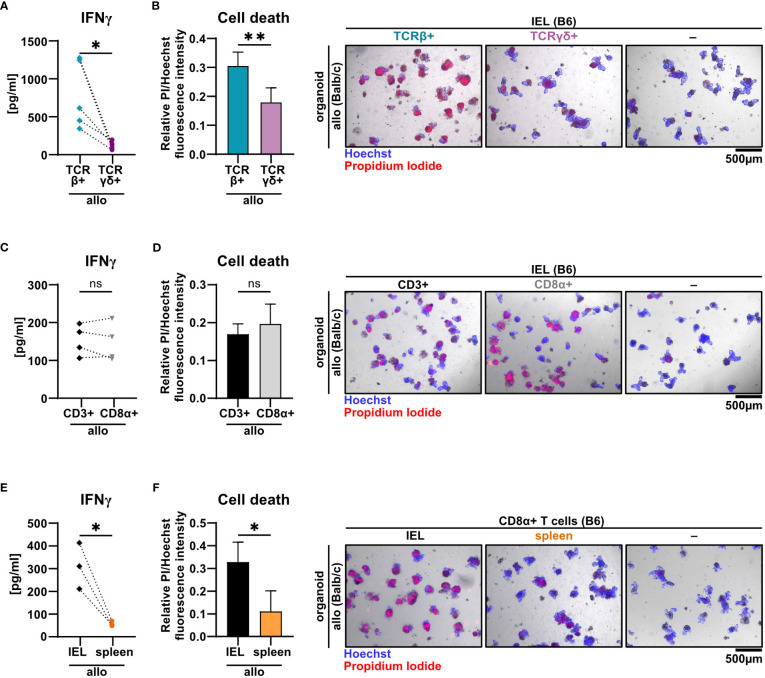
TCRβ+ CD8α+ T cells within SI IELs largely account for allo-T cell-mediated organoid cell death *ex vivo*. Balb/c (allo) SI organoids were co-cultured alone (-) or with 2.5 x 10^5^ T cells from C57BL/6 (B6) donor mice magnetically enriched for the subsets as indicated below. On d2 after start of the co-culture, cell-free supernatants were analyzed by ELISA and fluorometric cell death quantification was performed. For cell death quantification, the ratio of PI/Hoechst fluorescence intensities was corrected for the baseline cell death in control organoid cultures without T cells. **(A, B)** SI Balb/c organoids were co-cultured with allogeneic SI IELs from B6 donors enriched for TCRβ^+^ and TCRγδ^+^ resp. Shown results reflect data from n = 5 independent experiments. **(A)** IFNγ protein quantification in cell-free supernatant by ELISA. **(B)** Organoid cell death assessment (left panel) as determined by fluorometric analysis. On the right panel, representative images of organoids stained with Hoechst and PI after co-culture are displayed. **(C, D)** Data from n = 4 independent co-culture experiments consisting of SI Balb/c organoids and allogeneic B6 SI IELs enriched for CD3^+^ or CD8α^+^ T cells are displayed. In **(C)** IFNγ protein was quantified by ELISA in the cell-free supernatant at d2 of co-culture. **(D)** Fluorometric quantification of cell death (left panel) among organoids on d2 of co-culture is shown. In the right panel, representative microscopic images of organoids double-stained with Hoechst and PI after co-culture are displayed. **(E, F)** Results from allogeneic co-cultures of SI organoids from Balb/c mice with CD8α^+^-enriched IELs vs. CD8α^+^-enriched splenocytes from B6 mice are displayed. Depicted data were derived from d2 analyses and are pooled from n = 3 experiments. In **(E)**, IFNγ protein levels in the cell-free supernatant were determined by ELISA. In **(F)**, fluorometric cell death quantification (left panel) was performed. Representative microscopic images (right panel) of organoids double-stained with Hoechst and PI after co-culture are depicted. Graphs show pooled data from independent experiments (n = 3-5 as indicated), graphs **(B, D, F)** depict mean ± SD. ns (not significant) p > 0.05, *p  ≤ 0.05, **p ≤ 0.01, by two-tailed unpaired t test or t test with Welch’s correction [graphs **(A)** and **(E)**].

T cells residing within the SI IEL compartment comprise both TCRαβ^+^ and TCRγδ^+^ T cells. Interestingly, the TCRαβ/TCRγδ T cell ratio did not differ after the 2d co-culture time period between the allogeneic and syngeneic setting (**Supplementary Figures 3A, B**). Moreover, within the TCRαβ^+^ T cell pool, both CD4^+^, CD8αα^+^ and CD8αβ^+^ T cell subsets were virtually unaltered (**Supplementary Figure 3C**). To further elucidate which T cell subsets among IELs largely account for allo-T cell-mediated IEC death, we utilized TCRαβ^+^ and TCRγδ^+^ T cell-enriched IEL pools and assessed their behavior in the allogeneic co-culture model system. Firstly, we found that TCRγδ^+^ IELs expressed significantly less IFNγ compared to TCRαβ^+^ IEL pools (**Figure 5A**). Moreover, TCRαβ^+^ IELs were superior in mediating IEC death in comparison to TCRγδ^+^ T cells (**Figure 5B**). Secondly, CD8α^+^ T cells expressed IFNγ and executed IEC death equally efficient as the total, unfractionated CD3^+^ IEL pool suggesting that within the IEL-residing TCRαβ^+^ T cell fraction, CD8α^+^ were sufficient, while CD4^+^CD8α^−^ T cell subsets were dispensable for the effect observed in the allogeneic setting (**Figures 5C, D**). Finally, we confirmed that these characteristics were unique properties of bona fide IEL-derived CD8α^+^ T cells, as splenic CD8α^+^ T cells failed to comparably mount elevated IFNγ levels and mediate IEC death (**Figures 5E, F**). Together, small intestinal TCRαβ^+^ CD8α^+^ IELs largely account for the death of allogeneic organoids in our *ex vivo* co-culture model system.

### 3.6 Presence of minor mismatch antigens is sufficient to drive allo-IEL-mediated IEC death

**Figure 6:**
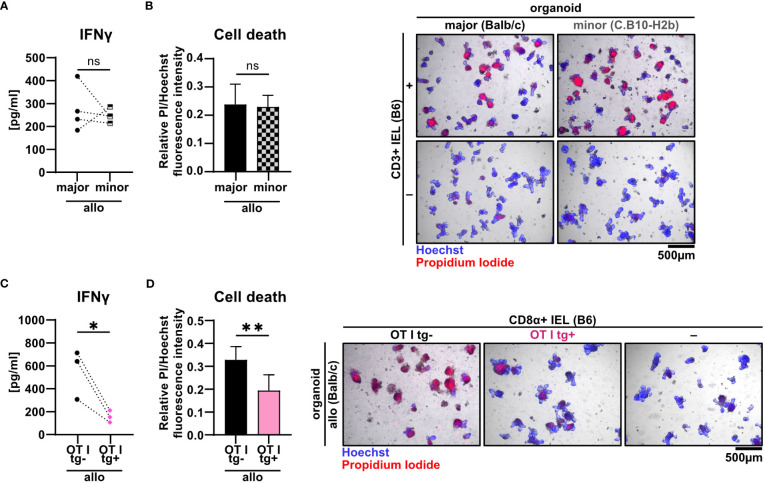
Minor mismatch antigens are sufficient for the CD3^+^ IEL-mediated killing of allogeneic IECs *ex vivo*. **(A, B)** 2.5 x 10^5^ C57BL/6 (B6) IELs enriched for CD3^+^ T cells were co-cultured with SI Balb/c organoids (allogeneic major mismatch, i.e. MHC I-mismatched) vs. SI organoids from C.B10-H2 b/LilMcdJ mice (allogeneic minor mismatch, i.e. MHC I-matched, minor histocompatibility antigen mismatched). Organoids cultured w/o T cells served as controls (-). **(A)** IFNγ protein quantification within cell-free supernatants was performed *via* ELISA at d2; **(B)** fluorometric organoid cell death quantification corrected for the organoid cell death observed in wells with organoids cultured w/o T cells (-) (left panel) and representative microscopic images of organoids stained with Hoechst/PI dyes after 2 days of co-culturing are displayed (right panel). Data represent pooled results from n = 4 independent experiments and depict mean ± SD. Data were analyzed by two-tailed unpaired t test. **(C, D)** Balb/c (allo) SI organoids were co-cultured with 2.5 x 10^5^ CD8α^+^-enriched IELs from OTI tg^+^ donor mice or OTI tg^-^ control mice (B6) or cultured w/o T cells (-) for 2d. Graphs show data from independent experiments as indicated and display **(C)** IFNγ protein quantification within cell-free supernatants performed *via* ELISA at d2 (n = 3); **(D)** organoid cell death after co-culture corrected for baseline organoid death detectable in wells culturing organoids alone, i.e. w/o T cells (-) (left panel, n = 7) as well as representative images of Hoechst and PI dye double-stained organoids (right panel). Data shown in **(D)** represent mean ± SD. ns (not significant) p > 0.05, *p  ≤ 0.05, ** p  ≤ 0.01 by two-tailed unpaired t test.

Current pre-clinical models of acute GvHD are either based on a complete mismatch of the major histocompatibility complex (MHC) between host and recipient or on minor histocompatibility antigen (miHA) mismatches alone. Therefore, we aimed to unravel the pathomechanistic requirements underlying allo-IEL mediated IEC death by investigating whether our finding of CD3^+^ IEL-mediated killing of organoids in a complete MHC mismatch constellation can be recapitulated in a minor mismatch setting *ex vivo*. To achieve this goal, we assayed both killing abilities and IFNγ expression levels of allogeneic IELs cultured either within organoids derived from C.B10-H2b/LilMcdJ mice (minor mismatch) or Balb/c mice (major mismatch). The C.B10-H2b/LilMcdJ strain genetically matches Balb/c mice with the exception that the endogenous MHC complex of Balb/c mice, H2d, has been replaced by H2b, equivalent to the MHC complex of B6 mice. Consequently, tissues derived from this line and B6 mice derived T cells are MHC-matched (H2b) but display a complete mismatch in miHA (33). As shown in **Figures 6A, B**, both IFNγ protein expression levels and IEC death rates were virtually identical between miHA and MHC mismatch settings *ex vivo*. Given our finding that mismatch in the miHA peptidome is sufficient, we finally wondered whether conversely the TCR specificity is critical for allo-IEL activation as determined by IFNγ release and IEL-mediated cytotoxicity within our model system. To address that question experimentally, we restricted the TCR specificity within the IEL CD8^+^ T cell pool by making use of a transgenic mouse line in which T cells carry a distinct T cell receptor for murine *Tcra-V2* and *Tcrb-V5* genes recognizing an immuno-dominant peptide of ovalbumin. Strikingly, CD8α^+^ T cells isolated from the SI IEL compartment of OTI transgenic (tg^+^) mice failed to mount IFNγ levels comparable to T cells with an endogenous, polyclonal TCR repertoire (OTI tg^-^) (**Figure 6C**). Consecutively, OTI tg^+^ CD8α^+^ T cells also elicited significantly less cytotoxicity towards IECs in the allogeneic setting compared to non-transgenic IELs (**Figure 6D**). In summary, allogeneic IEC killing requires specific antigen recognition of miHA by IELs independent of a complete MHC mismatch.

### 3.7 Allogeneic IELs display a restricted patrolling behavior within SI organoids *ex vivo*

**Figure 7:**
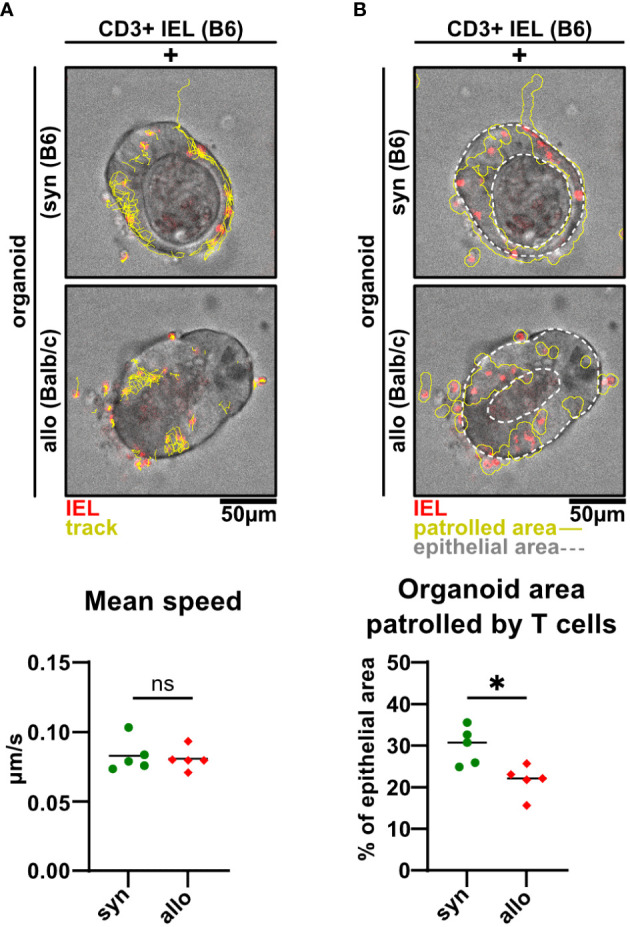
Alloreactive intraepithelial T cells display a more restricted patrolling behavior compared to syngeneic controls. **(A, B)** 2.5 x 10^5^ CD3^+^-enriched C57BL/6 (B6) SI IELs were fluorescently labeled with Proliferation Dye eFluor 670 and co-cultured within SI Balb/c organoids for 2d. Representative images and quantitative analyses from live cell imaging time-lapse studies at d2 of co-culturing are displayed. Time series were recorded on a spinning disc microscope with a 25X objective every 30 sec for a period of 45 min on d1 of co-culture. Analyses were performed using Fiji software and the TrackMate plugin as well as an in-house macro script. In **(A)** visualized tracks (yellow) of the movements of T cells (red) within intestinal organoids (top and middle panel) and quantification of their mean speed in μm/s as identified by the TrackMate plugin are shown (bottom panel). In **(B)** organoid area cumulatively covered by migrating IELs (red) over time is depicted (solid, yellow line) while the region of interest is indicated by white dotted lines (top and middle panel). Patrolled areas were quantified by relating this region to the region of interest as determined using an in-house Fiji macro. Graphs show data from n = 5 independent experiments; n = 4 technical replicates per group were recorded in each experiment. ns (not significant) p > 0.05, *p  ≤ 0.05 by two-tailed unpaired t test.

As previously demonstrated for the syngeneic setting (19), live cell imaging of IEL/IEC co-cultures *ex vivo* offers the opportunity to visualize IEL migratory properties under defined conditions over time. Here, we sought to apply this methodology to decipher whether allogeneic and syngeneic IELs display differential migratory properties. As shown in **Figure 7A** and **Supplementary Video 2**, we found IELs to be patrolling SI organoids with a comparable speed irrespective of an allogeneic or syngeneic setting. However, the organoid area scanned by migrating allo-IELs turned out to be significantly smaller compared to syngeneic controls (**Figure 7B**). Overall, limited area coverage by allo-IELs within organoids might indicate that upon antigen-recognition, defined clones are locally restrained and then exert IEC cytotoxicity as their default functional assignment.

## 4 Discussion

Intestinal GvHD remains a major challenge in the management of allo-HSCT patients. While donor T cells are acceptedly critical mediators of allogeneic intestinal inflammation, selectively T cell-targeting therapies have not been developed yet despite an urgent medical need for innovative treatment options. One underlying cause represents sparse knowledge on T cell subset-specific roles and here especially insight into their impact on the target tissue mostly affected in the course of GI GvHD, the epithelial layer. Interestingly, some early studies have reported that the intestinal epithelial layer is routinely populated by immune cells, so called IELs, predominately composed of T cells, suggesting that IELs may directly exert IEC damaging effects (14–16). Despite this observation, the behavior within and specific impact of donor T cells on IECs have been poorly explored so far. This is in part due to methodological limitations arising when IELs are studied *in vivo* or *ex vivo* (17, 20). To overcome this shortcoming, we sought to employ a novel IEL/IEC co-culture model system recently reported by us and others as a valuable tool to study IEL function and behavior within syngeneic small intestinal organoids *ex vivo* (17, 19, 20). In this study, we applied this experimental model system to the allogeneic context with the goal to provide a model system that will help to fill this methodological and biological gap, as it enables future studies to explore the lympho-epithelial crosstalk in an allogeneic setting *ex vivo* by mimicking aspects of intestinal GvHD *in vivo*.

Employing our *ex vivo* co-culture model, we found that allogeneic SI IELs readily populated SI organoids while we were unable to detect a noticeable regulation of the IEL composition between syngeneic and allogeneic culture conditions. Of note, we observed significantly elevated IL-2 protein expression levels pointing at robust T cell activation, as IL-2 expression represents a classical landmark of T cell activation downstream of TCR engagement (34), as well as strongly up-regulated IFNγ expression within allogeneic co-cultures. This is in line with previous reports identifying IL-2 as a strong inducer of IFNγ expression and cytotoxic modules within T cells (35, 36).

In delineation from the syngeneic setting, however, our studies were limited to two days as we noticed at this time point significant changes in cell viability in the allogeneic co-cultures: Importantly, we could show that allogeneic IELs induce significantly higher cell death levels in co-culture compared to syngeneic IELs. Still, we recorded elevated cell death rates also in syngeneic co-cultures when compared to spontaneous, basal cell death events in B6- or Balb/c-derived organoids alone, i.e. in the absence of T cells. As shown by others and our functional studies using IFNγ-deficient IELs, at this point we attribute reduced organoid viability in the presence of syngeneic IELs to the presence of T cell-derived IFNγ. Multiple scenarios may apply to explain this finding: At first, subtle, not traceable genetic differences among different offspring of syngeneic inbred strains serving as B6 IEL and B6 organoid resp. donor mice may be sufficient to trigger T cell activation and concomitant IFNγ expression. Alternatively, IECs that undergo routine shedding upon IEC renewal are *in vivo* physiologically shed to the lumen and are discarded by the stool flow while in our *ex vivo* organoid system they stay *in situ.* Hence, IEC-derived antigens and danger signals e.g. from carried over microbiota might elicit syngeneic IEL activation. Importantly, our characterization of allogeneic IEL-mediated effects on organoids did not only reveal enhanced IEC death but also evidence for a negative regulation of the intestinal stem cell niche given a significant reduction of the expression levels of intestinal stem cell markers Lgr5 and Olfm4 in allogeneic compared to syngeneic conditions. Moreover, and in line with recently published studies, IFNγ expressed by alloreactive IEL T cells turned out to be critical for the abrogated stem cell homeostasis, as IFNγ-deficient IEL T cells did not only elicit reduced cell death but also failed to diminish Lgr5 and Olfm4 expression levels (31, 37). Hence, morphological and molecular characteristics of allogeneic IEL/IEC co-cultures identified by our study overall display aspects that are reminiscent of findings in human intestinal GvHD and highlights IFNγ^+^ IELs as putatively critical mediators in its pathogenesis (31, 37).

Our studies clearly uncovered that allogeneic T cells isolated from the SI IEL compartment exert cytotoxic effects on IEC that are seemingly unique, as lymphoid-resident spleen-derived T cells were unable to mimic this function. This may be explained by largely unexplored functional properties of IEL T cells compared to splenic T cells somehow adapted to the epithelial environment. However, another obvious argument to explain these cell-type restricted abilities is that IELs largely consist of antigen-experienced, so called tissue-resident memory (Trm) cells that are able to readily exert their default functions upon TCR triggering while splenic T cell pools mainly comprise antigen-unexperienced, naïve cell types requiring additional signals for activation, priming and overall functionality (13, 38, 39). Within the IEL T cell subsets, we found that in fact TCRαβ^+^ were superior to TCRγδ^+^ T cells with regard to IFNγ expression and IEC killing abilities in an allogeneic setting. Many studies on their contribution are inconclusive. While there are recently emerging indications for functionally relevant, context-dependent anti-tumoral and anti-leukemic, cytotoxic effects of TCRγδ^+^ T cells (40, 41), the majority of reports suggests rather immune-regulatory than inflammation-promoting effects of TCRγδ^+^ T cells during intestinal GvHD (42–44). Therefore, our finding of a seemingly minor role for IEL TCRγδ^+^ T cells in mediating immune-mediated IEC death in the allogeneic setting is in line with the current state of the field.

Moreover, we found that within TCRαβ^+^ IELs CD4^+^CD8α^-^ T cells are seemingly dispensable for exerting IEC death as IEL CD8α^+^ T cells lacking CD4^+^CD8α^-^ T cells compared to total CD3^+^ T cells displayed virtually indistinguishable effects albeit there was a trend that the former showed more pronounced cytoxicity. This is in line with recently emerging evidence suggesting that Tregs residing predominately in the lamina propria are able to convert into CD4^+^CD8α^-^ intra-epithelial T cells which then exert intestinal inflammation-suppressive functions (45). Future studies, however, will have to determine whether CD4^+^CD8α^-^ intra-epithelial T cells are exerting allogeneic IEL-driven IEC death-reducing and intestinal stem cell compartment-protecting effects. Thus, our data show that the CD8α^+^ Trm population within IEL TCRαβ^+^ T cells primarily accounts for the cytotoxic effects affecting SI organoids. Importantly, this is in agreement with reports by others showing that donor T cell populations infiltrating the intestinal epithelia during GvHD express transcriptional programs and surface markers (e.g. CD103) characteristic for CD8α^+^ Trm T cells (46–48).

Having functionally defined the cellular components, we sought to further elucidate the mechanistic cues contributing to IEL-mediated IEC death in our co-culture model system. By additionally applying major and minor mismatch conditions onto the allogeneic setting, we found that major mismatch, i.e. a full MHC mismatch between IEL and organoids, is not a condition sine qua non in our IEL/IEC co-culture model system: We strikingly found that both IFNγ secretion and IEC killing by allogeneic IELs were virtually indistinguishable between MHC-mismatched (major) and MHC-matched (minor, i.e. miHA mismatched) conditions, pointing at the critical role of the presented peptide in this setting.

Over the last decades, alternative pathogenetic concepts have been developed to mechanistically explain direct allorecognition of foreign target cells by T cells. Strikingly, the frequency of alloreactive T cells within a given MHC-mismatched donor is approximately between 1-10%, which is exceptionally high compared to the frequency of T cells specific for a given MHC-peptide complex (49, 50). In the light of this finding, the so-called MHC-centric model suggests that polymorphisms in amino acids between self- and allo-MHC molecules affect the manner in which individual TCRs bind to MHC molecules. In this model, the presented peptide itself only marginally contributes to the recognition process, yielding a high frequency of T cells recognizing the allogeneic MHC molecule with various affinities (50, 51). As opposed to this, the peptide-centric hypothesis presumes that alloreactive T cells are specifically recognizing allopeptides presented on MHC receptors, a model system that is supported by the fact that alloreactive T cell populations are in fact highly peptide specific (50–52). Since both models are supported by functional and structural data, realistically, in most interactions probably both the MHC molecule as well as the peptide contribute to T cell recognition depending on the specific context.

To further assess the relative contribution of the MHC/peptide complex composition on the activation and functionality of allogeneic IELs, we employed OT I transgenic mice on the B6 genetic background carrying a genetically modified, non-endogenous TCR as the source of allogeneic IELs. OT I^+^ T cells express a TCR that only detects a peptide derived from ovalbumin, i.e. an exogenous antigen that is not present in our co-culture model system. Importantly, CD8α^+^ OT I tg^+^ IELs failed to mount an alloresponse (i.e. IFNγ expression and IEC death induction) in the presence of MHC-mismatched organoids. As the employed OT I transgenic mouse model represented mice not previously back-crossed on a RAG-1/2 deficient background, our data show that OT I tg^+^ T cells despite containing a minor T cell pool expressing a residual endogenous TCR repertoire are hampered in their ability to induce allo-mediated cell death, indicating that a sufficiently high number and/or diversity of TCR repertoire is required to elicit a robust allo-response in our model system. Together with results derived from the major vs. minor mismatch setting, the shown data suggest that allogeneic IEL activation and functionality rather depend on the TCR recognition of the presented, peptide-derived epitope than alone on the (mismatched) MHC molecule itself. Based on these results, we conclude at this point that allogeneic IELs isolated from B6 donors recognize endogenous peptides on IEC characteristic for the Balb/c genetic background. Our study is certainly limited as it does not ultimately resolve this critical question. Regardless, the IEL/IEC co-culture model system and our initial characterization have certainly laid the methodological basis for more in-depth investigations of the assumed allo-specificity of intestinal IELs in the future.

A major population of IEL T cells express the integrin αEβ7 (CD103) which is known to bind E-Cadherin on IEC thereby presumably conferring IEL tissue retention (10, 39). Moreover, we described earlier that CD103 seems to be a critical component of normal IEL migratory properties within intestinal epithelia, at least *ex vivo* (11, 12). So far, however, the exact mechanisms regulating IEL migration and motility within the epithelial layer both in the steady state and under inflammatory conditions including intestinal GvHD remain only incompletely understood.

One group investigating T cell localization in the context of GvHD-mediated intestinal damage by 3D microscopy found that donor T cells preferentially accumulated in the lower crypt region after allo-HSCT (53). In this study a direct interaction between T cells and intestinal stem cells was described and associated to the occurrence of damaged crypts already 4 days after transplantation, indicating that donor T cell population of the IEL compartment is an early event in GvHD pathology and intestinal stem cells are a primary target of allogeneic T cells (53). Hence, novel experimental platforms and modalities like the IEL/IEC allogeneic co-culture model characterized in our study provide the unique opportunity to further elucidate the lympho-epithelial interaction as e.g. migration also in a spatiotemporal and functional manner *ex vivo*. Therefore, we applied our previously reported live cell imaging microscopy protocol on the allogeneic setting (19). Strikingly, we found that allogeneic T cells displayed a regionally more restricted patrolling behavior contrasting syngeneic T cells covering larger areas within organoids over time. Currently, we cannot provide an analysis with a higher spatiotemporal resolution to e.g. determine whether this is due to the fact that allogeneic IEL T cells orientate themselves *ex vivo* towards the base of the crypts and stem cell niche as described for the *in vivo* behavior (53). Regardless, considering our finding that allogeneic IELs are clearly dependent on allo-peptide recognition and are more strongly activated as evidenced by elevated IFNγ expression levels, for us one explanation model is that upon antigen recognition, IELs may stay more locally confined to exert their cytotoxic effector functions more focused towards their target cells. This is in line with the observation by another group (20), who qualitatively described that OT I tg^+^ IELs in syngeneic co-culture with small intestinal organoids seemed to arrest in their movement when Ova peptide was added, i.e. upon TCR engagement. However, future studies directly focusing on this aspect will be required but will definitely benefit from the availability of our allogeneic IEL/IEC co-culture model system.

Finally, in delineation from other published studies using allogeneic T cell/organoid co-culture model systems (31, 54, 55), we omitted *in vivo* (e.g. in the course of allo-HSCT) or in a way artificial, broad *ex vivo* activation (e.g. mimicking antigen receptor signaling through PMA/ionomycine or anti-CD3/anti-CD28 stimulation) of the T cells prior to exposition to IECs. By doing so, we focus on the spontaneous, natural activation pattern of alloreactive T cells by epithelial cell structures. In fact, by showing that unstimulated splenic T cells were deficient in inducing IEC death in our co-culture setting, we could demonstrate that mounting a cytotoxic allogeneic response without the requirement of additional (co-)activation is a unique feature of IELs. Hence, our observations strongly indicate that IELs as bona fide antigen-experienced T cells possess reactivity to antigens presented in an allogeneic setting. Importantly, IELs are responsive to those antigen pools upon encountering them in their home tissue without requiring additional priming or signaling by APCs in lymph nodes.

As a limitation, our co-culture system only partially reflects critical aspects closely associated with *in vivo* GvHD pathology. For example, the contribution of innate immune cells such as granulocytes, antigen-presenting cells and ILCs all known to be able to modulate intestinal inflammation in GvHD, is omitted (56). In addition, the role of fungal commensals and enteric viruses, such as CMV reactivation as a common complication in patients previously undergoing allo-HSCT, is not part of the current model system and remains to be integrated in the future (57, 58). Furthermore, IEL/IEC co-cultures are devoid of luminal signals, especially from the intestinal microbiota. Given the nowadays widely accepted, central impact of microbial signals on the initiation and course of intestinal GvHD (59), future attempts to develop this model system further will include the evaluation of modalities allowing the inclusion of both microbiota-derived inflammatory signals and microbial peptides. Lastly, it represents a valuable future goal to apply this model to the human setting, as it has already been achieved successfully for *ex vivo* autologous co-cultures of intestinal T cells and organoids from patients with inflammatory bowel disease (60). Ultimately, the translation to the human system would allow to investigate the IEL/IEC interaction in a more clinical and patient-centered manner.

In summary, our here presented *ex vivo* model system allows multimodal functional studies to investigate the specific regulation of IECs by intestinal allogeneic IEL T cells, thereby overall displaying multiple characteristics reminiscent of intestinal GvHD pathophysiology. We strongly believe that the use of this allogeneic IEL/organoid co-culture model system will facilitate studies designed to elucidate allogeneic pathomechanisms driven by donor-derived IEL T cells with the ultimate goal to pave the way for the identification of novel therapeutic strategies and target structures.

## Data availability statement

The original contributions presented in the study are included in the article/**Supplementary Material**. Further inquiries can be directed to the corresponding author.

## Ethics statement

The animal study was approved by government of Lower and Middle Franconia. The study was conducted in accordance with the local legislation and institutional requirements.

## Author contributions

DM: Conceptualization, Data curation, Formal Analysis, Investigation, Methodology, Validation, Writing – original draft, Writing – review & editing. MD: Methodology, Resources, Writing – review & editing. BS: Data curation, Methodology, Resources, Software, Writing – review & editing. TV: Investigation, Methodology, Project administration, Resources, Writing – review & editing. MN: Conceptualization, Resources, Supervision, Writing – review & editing. HP: Methodology, Resources, Writing – review & editing. CN: Methodology, Resources, Supervision, Writing – review & editing. MB-H: Conceptualization, Funding acquisition, Investigation, Methodology, Supervision, Writing – review & editing. KH: Conceptualization, Data curation, Formal Analysis, Funding acquisition, Methodology, Project administration, Resources, Supervision, Validation, Visualization, Writing – original draft, Writing – review & editing.

## References

[1] Copelan E Casper JT Carter SL van Burik JA Hurd D Mendizabal AM. A scheme for defining cause of death and its application in the T cell depletion trial. Biol Blood Marrow Transplant (2007) 13(12):1469–76. doi: 10.1016/j.bbmt.2007.08.047 18022577

[2] Zeiser R Blazar BR. Acute graft-versus-host disease - biologic process, prevention, and therapy. N Engl J Med (2017) 377(22):2167–79. doi: 10.1056/NEJMra1609337 PMC6034180 29171820

[3] Jaksch M Mattsson J. The pathophysiology of acute graft-versus-host disease. Scand J Immunol (2005) 61(5):398–409. doi: 10.1111/j.1365-3083.2005.01595.x 15882431

[4] Choi SW Levine JE Ferrara JL. Pathogenesis and management of graft-versus-host disease. Immunol Allergy Clin North Am (2010) 30(1):75–101. doi: 10.1016/j.iac.2009.10.001 20113888 PMC4141413

[5] Ara T Hashimoto D. Novel insights into the mechanism of GVHD-induced tissue damage. Front Immunol (2021) 12:713631. doi: 10.3389/fimmu.2021.713631 34512636 PMC8429834

[6] Jansen SA Nieuwenhuis EES Hanash AM Lindemans CA. Challenges and opportunities targeting mechanisms of epithelial injury and recovery in acute intestinal graft-versus-host disease. Mucosal Immunol (2022) 15(4):605–19. doi: 10.1038/s41385-022-00527-6 PMC9259481 35654837

[7] Harris AC Young R Devine S Hogan WJ Ayuk F Bunworasate U. International, multicenter standardization of acute graft-versus-host disease clinical data collection: A report from the mount sinai acute GVHD international consortium. Biol Blood Marrow Transplant (2016) 22(1):4–10. doi: 10.1016/j.bbmt.2015.09.001 26386318 PMC4706482

[8] Naymagon S Naymagon L Wong SY Ko HM Renteria A Levine J. Acute graft-versus-host disease of the gut: considerations for the gastroenterologist. Nat Rev Gastroenterol Hepatol (2017) 14(12):711–26. doi: 10.1038/nrgastro.2017.126 PMC6240460 28951581

[9] Ma H Qiu Y Yang H. Intestinal intraepithelial lymphocytes: Maintainers of intestinal immune tolerance and regulators of intestinal immunity. J Leukoc Biol (2021) 109(2):339–47. doi: 10.1002/JLB.3RU0220-111 PMC7891415 32678936

[10] Cheroutre H Lambolez F Mucida D. The light and dark sides of intestinal intraepithelial lymphocytes. Nat Rev Immunol (2011) 11(7):445–56. doi: 10.1038/nri3007 PMC3140792 21681197

[11] Masopust D Choo D Vezys V Wherry EJ Duraiswamy J Akondy R. Dynamic T cell migration program provides resident memory within intestinal epithelium. J Exp Med (2010) 207(3):553–64. doi: 10.1084/jem.20090858 PMC2839151 20156972

[12] Cheng L Becattini S. Intestinal CD8(+) tissue-resident memory T cells: From generation to function. Eur J Immunol (2022) 52(10):1547–60. doi: 10.1002/eji.202149759 PMC9804592 35985020

[13] McDonald BD Jabri B Bendelac A. Diverse developmental pathways of intestinal intraepithelial lymphocytes. Nat Rev Immunol (2018) 18(8):514–25. doi: 10.1038/s41577-018-0013-7 PMC6063796 29717233

[14] Tsuzuki T Yoshikai Y Ito M Mori N Ohbayashi M Asai J. Kinetics of intestinal intraepithelial lymphocytes during acute graft-versus-host disease in mice. Eur J Immunol (1994) 24(3):709–15. doi: 10.1002/eji.1830240333 7907297

[15] Schattenfroh NC Hoffman RA McCarthy SA Simmons RL. Phenotypic analysis of donor cells infiltrating the small intestinal epithelium and spleen during graft-versus-host disease. Transplantation. (1995) 59(2):268–73. doi: 10.1097/00007890-199501000-00020 7839451

[16] Nussler NC Hoffman RA McCarthy SA Simmons RL. Functional changes of intestinal intraepithelial lymphocytes during acute graft versus host disease: correlation with phenotype. Int Immunol (1996) 8(11):1767–77. doi: 10.1093/intimm/8.11.1767 8943572

[17] Nozaki K Mochizuki W Matsumoto Y Matsumoto T Fukuda M Mizutani T. Co-culture with intestinal epithelial organoids allows efficient expansion and motility analysis of intraepithelial lymphocytes. J Gastroenterol (2016) 51(3):206–13. doi: 10.1007/s00535-016-1170-8 PMC4771822 26800996

[18] Sato T Vries RG Snippert HJ van de Wetering M Barker N Stange DE. Single Lgr5 stem cells build crypt-villus structures in *vitro* without a mesenchymal niche. Nature. (2009) 459(7244):262–5. doi: 10.1038/nature07935 19329995

[19] Enderle K Dinkel M Spath EM Schmid B Zundler S Tripal P. Dynamic imaging of IEL-IEC co-cultures allows for quantification of CD103-dependent T cell migration. Int J Mol Sci (2021) 22(10). doi: 10.3390/ijms22105148 PMC8152227 34067987

[20] Rogoz A Reis BS Karssemeijer RA Mucida D. A 3-D enteroid-based model to study T-cell and epithelial cell interaction. J Immunol Methods (2015) 421:89–95. doi: 10.1016/j.jim.2015.03.014 25841547 PMC4451438

[21] Ullrich E Abendroth B Rothamer J Huber C Buttner-Herold M Buchele V. BATF-dependent IL-7RhiGM-CSF+ T cells control intestinal graft-versus-host disease. J Clin Invest (2018) 128(3):916–30. doi: 10.1172/JCI89242 PMC5824870 29376889

[22] Buchele V Abendroth B Buttner-Herold M Vogler T Rothamer J Ghimire S. Targeting Inflammatory T Helper Cells *via* Retinoic Acid-Related Orphan Receptor Gamma t Is Ineffective to Prevent Allo-Response-Driven Colitis. Front Immunol (2018) 9:1138. doi: 10.3389/fimmu.2018.01138 29910804 PMC5992389

[23] Bode KJ Mueller S Schweinlin M Metzger M Brunner T. A fast and simple fluorometric method to detect cell death in 3D intestinal organoids. Biotechniques. (2019) 67(1):23–8. doi: 10.2144/btn-2019-0023 31218886

[24] Schmid B Tripal P Fraass T Kersten C Ruder B Gruneboom A. 3Dscript: animating 3D/4D microscopy data using a natural-language-based syntax. Nat Methods (2019) 16(4):278–80. doi: 10.1038/s41592-019-0359-1 30886414

[25] Enderle K Dinkel M Spath EM Schmid B Zundler S Tripal P. Dynamic imaging of IEL-IEC co-cultures allows for quantification of CD103-dependent T cell migration. Int J Mol Sci (2021) 22(10). doi: 10.3390/ijms22105148 PMC8152227 34067987

[26] Linkert M Rueden CT Allan C Burel JM Moore W Patterson A. Metadata matters: access to image data in the real world. J Cell Biol (2010) 189(5):777–82. doi: 10.1083/jcb.201004104 PMC2878938 20513764

[27] Tinevez JY Perry N Schindelin J Hoopes GM Reynolds GD Laplantine E. TrackMate: An open and extensible platform for single-particle tracking. Methods. (2017) 115:80–90. doi: 10.1016/j.ymeth.2016.09.016 27713081

[28] Barker N van Es JH Kuipers J Kujala P van den Born M Cozijnsen M. Identification of stem cells in small intestine and colon by marker gene Lgr5. Nature. (2007) 449(7165):1003–7. doi: 10.1038/nature06196 17934449

[29] van der Flier LG Haegebarth A Stange DE van de Wetering M Clevers H. OLFM4 is a robust marker for stem cells in human intestine and marks a subset of colorectal cancer cells. Gastroenterology. (2009) 137(1):15–7. doi: 10.1053/j.gastro.2009.05.035 19450592

[30] Wang H Asavaroengchai W Yeap BY Wang MG Wang S Sykes M. Paradoxical effects of IFN-gamma in graft-versus-host disease reflect promotion of lymphohematopoietic graft-versus-host reactions and inhibition of epithelial tissue injury. Blood. (2009) 113(15):3612–9. doi: 10.1182/blood-2008-07-168419 PMC2668848 19211507

[31] Takashima S Martin ML Jansen SA Fu Y Bos J Chandra D. T cell-derived interferon-gamma programs stem cell death in immune-mediated intestinal damage. Sci Immunol (2019) 4(42). doi: 10.1126/sciimmunol.aay8556 PMC7239329 31811055

[32] Chavez-Galan L Arenas-Del Angel MC Zenteno E Chavez R Lascurain R. Cell death mechanisms induced by cytotoxic lymphocytes. Cell Mol Immunol (2009) 6(1):15–25. doi: 10.1038/cmi.2009.3 19254476 PMC4002546

[33] Freedman HA Lilly F. Properties of cell lines derived from tumors induced by Friend virus in BALB/c and BALB/c-H-2b mice. J Exp Med (1975) 142(1):212–23. doi: 10.1084/jem.142.1.212 PMC2189885 1056976

[34] Jain J Loh C Rao A. Transcriptional regulation of the IL-2 gene. Curr Opin Immunol (1995) 7(3):333–42. doi: 10.1016/0952-7915(95)80107-3 7546397

[35] Reem GH Yeh NH. Interleukin 2 regulates expression of its receptor and synthesis of gamma interferon by human T lymphocytes. Science. (1984) 225(4660):429–30. doi: 10.1126/science.6429853 6429853

[36] Janas ML Groves P Kienzle N Kelso A. IL-2 regulates perforin and granzyme gene expression in CD8+ T cells independently of its effects on survival and proliferation. J Immunol (2005) 175(12):8003–10. doi: 10.4049/jimmunol.175.12.8003 16339537

[37] Eriguchi Y Nakamura K Yokoi Y Sugimoto R Takahashi S Hashimoto D. Essential role of IFN-gamma in T cell-associated intestinal inflammation. JCI Insight (2018) 3(18). doi: 10.1172/jci.insight.121886 PMC6237234 30232288

[38] Behr FM Chuwonpad A Stark R van Gisbergen K. Armed and ready: Transcriptional regulation of tissue-resident memory CD8 T cells. Front Immunol (2018) 9:1770. doi: 10.3389/fimmu.2018.01770 30131803 PMC6090154

[39] Mueller SN Mackay LK. Tissue-resident memory T cells: local specialists in immune defence. Nat Rev Immunol (2016) 16(2):79–89. doi: 10.1038/nri.2015.3 26688350

[40] Maniar A Zhang X Lin W Gastman BR Pauza CD Strome SE. Human gammadelta T lymphocytes induce robust NK cell-mediated antitumor cytotoxicity through CD137 engagement. Blood. (2010) 116(10):1726–33. doi: 10.1182/blood-2009-07-234211 PMC3324253 20519625

[41] de Vries NL van de Haar J Veninga V Chalabi M Ijsselsteijn ME van der Ploeg M. gammadelta T cells are effectors of immunotherapy in cancers with HLA class I defects. Nature. (2023) 613(7945):743–50. doi: 10.1038/s41586-022-05593-1 PMC9876799 36631610

[42] Minculescu L Marquart HV Ryder LP Andersen NS Schjoedt I Friis LS. Improved overall survival, relapse-free-survival, and less graft-vs.-host-disease in patients with high immune reconstitution of TCR gamma delta cells 2 months after allogeneic stem cell transplantation. Front Immunol (2019) 10:1997. doi: 10.3389/fimmu.2019.01997 31507601 PMC6714591

[43] Arruda LCM Gaballa A Uhlin M. Impact of gammadelta T cells on clinical outcome of hematopoietic stem cell transplantation: systematic review and meta-analysis. Blood Adv (2019) 3(21):3436–48. doi: 10.1182/bloodadvances.2019000682 PMC6855117 31714966

[44] Ye W Kong X Zhang W Weng Z Wu X. The roles of gammadelta T cells in hematopoietic stem cell transplantation. Cell Transplant (2020) 29:963689720966980. doi: 10.1177/0963689720966980 33073597 PMC7784584

[45] Sujino T London M Hoytema van Konijnenburg DP Rendon T Buch T Silva HM. Tissue adaptation of regulatory and intraepithelial CD4(+) T cells controls gut inflammation. Science. (2016) 352(6293):1581–6. doi: 10.1126/science.aaf3892 PMC4968079 27256884

[46] Tkachev V Kaminski J Potter EL Furlan SN Yu A Hunt DJ. Spatiotemporal single-cell profiling reveals that invasive and tissue-resident memory donor CD8(+) T cells drive gastrointestinal acute graft-versus-host disease. Sci Transl Med (2021) 13(576). doi: 10.1126/scitranslmed.abc0227 PMC9469805 33441422

[47] El-Asady R Yuan R Liu K Wang D Gress RE Lucas PJ. TGF-beta-dependent CD103 expression by CD8(+) T cells promotes selective destruction of the host intestinal epithelium during graft-versus-host disease. J Exp Med (2005) 201(10):1647–57. doi: 10.1084/jem.20041044 PMC2212926 15897278

[48] Liu K Anthony BA Yearsly MM Hamadani M Gaughan A Wang JJ. CD103 deficiency prevents graft-versus-host disease but spares graft-versus-tumor effects mediated by alloreactive CD8 T cells. PLoS One (2011) 6(7):e21968. doi: 10.1371/journal.pone.0021968 21779359 PMC3136479

[49] Abdelsamed HA Lakkis FG. The role of self-peptides in direct T cell allorecognition. J Clin Invest (2021) 131(21). doi: 10.1172/JCI154096 PMC8553548 34720090

[50] Boardman DA Jacob J Smyth LA Lombardi G Lechler RI. What is direct allorecognition? Curr Transplant Rep (2016) 3(4):275–83. doi: 10.1007/s40472-016-0115-8 PMC5107184 27909647

[51] Lechler RI Garden OA Turka LA. The complementary roles of deletion and regulation in transplantation tolerance. Nat Rev Immunol (2003) 3(2):147–58. doi: 10.1038/nri1002 12563298

[52] Felix NJ Allen PM. Specificity of T-cell alloreactivity. Nat Rev Immunol (2007) 7(12):942–53. doi: 10.1038/nri2200 18007679

[53] Fu YY Egorova A Sobieski C Kuttiyara J Calafiore M Takashima S. T cell recruitment to the intestinal stem cell compartment drives immune-mediated intestinal damage after allogeneic transplantation. Immunity. (2019) 51(1):90–103 e3. doi: 10.1016/j.immuni.2019.06.003 31278057 PMC7239328

[54] Matsuzawa-Ishimoto Y Hine A Shono Y Rudensky E Lazrak A Yeung F. An intestinal organoid-based platform that recreates susceptibility to T-cell-mediated tissue injury. Blood. (2020) 135(26):2388–401. doi: 10.1182/blood.2019004116 PMC7317146 32232483

[55] Göttert S Fischer JC Eisenkolb G Thiele Orberg E Jarosch S Holler E. IFN-gamma is crucial for the counterbalance of T cell-mediated injury to the intestinal stem cell compartment by regulatory T cells in mice and humans. Blood (2022) 140(Supplement 1):10223–4. doi: 10.1182/blood-2022-169303

[56] Peled JU Hanash AM Jenq RR. Role of the intestinal mucosa in acute gastrointestinal GVHD. Hematol Am Soc Hematol Educ Program (2016) 2016(1):119–27. doi: 10.1182/asheducation-2016.1.119 PMC5575743 27913470

[57] Cantoni N Hirsch HH Khanna N Gerull S Buser A Bucher C. Evidence for a bidirectional relationship between cytomegalovirus replication and acute graft-versus-host disease. Biol Blood Marrow Transplant (2010) 16(9):1309–14. doi: 10.1016/j.bbmt.2010.03.020 20353832

[58] Cho BS Yahng SA Kim JH Yoon JH Shin SH Lee SE. Impact of cytomegalovirus gastrointestinal disease on the clinical outcomes in patients with gastrointestinal graft-versus-host disease in the era of preemptive therapy. Ann Hematol (2013) 92(4):497–504. doi: 10.1007/s00277-012-1632-x 23180439

[59] Mathewson ND Jenq R Mathew AV Koenigsknecht M Hanash A Toubai T. Corrigendum: Gut microbiome-derived metabolites modulate intestinal epithelial cell damage and mitigate graft-versus-host disease. Nat Immunol (2016) 17(10):1235. doi: 10.1038/ni1016-1235b 27648549

[60] Hammoudi N Hamoudi S Bonnereau J Bottois H Perez K Bezault M. Autologous organoid co-culture model reveals T cell-driven epithelial cell death in Crohn's Disease. Front Immunol (2022) 13:1008456. doi: 10.3389/fimmu.2022.1008456 36439157 PMC9685428

