# Supplementary material for: Novel T cell/organoid culture system allows ex vivo modeling of intestinal graft-versus-host disease
Source: Front Immunol. 2023 Aug 29;14:1253514. doi: 10.3389/fimmu.2023.1253514 (PMC10495981; doi:10.3389/fimmu.2023.1253514)
Supplement: Supplementary file 1 [file DataSheet_1.pdf]

Supplementary Video Legends

Supplementary Video 1. Representative three-dimensional animation of cell death events within allogeneic and syngeneic IEL/organoid co-cultures and respective controls.
Syngeneic (C57BL/B6 (B6)) or allogeneic (Balb/c) organoids were co-cultured with 2.5 x 10^5 CD3+-enriched B6 SI IELs or without T cells as controls. T cells (green) were labeled with Proliferation Dye eFluor 670 prior to co-culturing, organoids were stained with Hoechst (blue) and PI (red) on d2 of co-culture. Z-stacks were taken with a confocal Leica SP5 microscope and reconstructed using Fiji software. The video shows an animation created with Fiji software and the plugin 3Dscript. Scalebars: 100 µm.

Supplementary Video 2. Time-lapse videos of syngeneic and allogeneic $CD3^+$ IEL migration in small intestinal organoids *ex vivo*.
2.5 x 10^5 $CD3^+$ C57BL/6 (B6) SI IELs were co-cultured with SI allogeneic (Balb/c) or syngeneic (B6) organoids. T cells were labeled with the Proliferation Dye eFluor 670 (here depicted in red) before co-culturing with indicated organoids. Time series were recorded on d1 of co-culture on a spinning disc microscope with a 25X objective for 45min with each frame taken 30 sec apart. Analyses were performed using Fiji software and the TrackMate plugin as well as an in-house macro script. The left panel shows time lapse series as recorded. In the middle panel, individual tracks of single IELs identified by the TrackMate plugin illustrating IEL migration within organoids are depicted by yellow lines. In the right panel, the overall area enclosed by migrating IELs as calculated by our in-house Fiji macro is illustrated by circular, solid yellow lines.
