# Supplementary material for: Novel T cell/organoid culture system allows ex vivo modeling of intestinal graft-versus-host disease
Source: Front Immunol. 2023 Aug 29;14:1253514. doi: 10.3389/fimmu.2023.1253514 (PMC10495981; doi:10.3389/fimmu.2023.1253514)
Supplement: Supplementary file 2 [file DataSheet_2.pdf]

Supplementary Figures

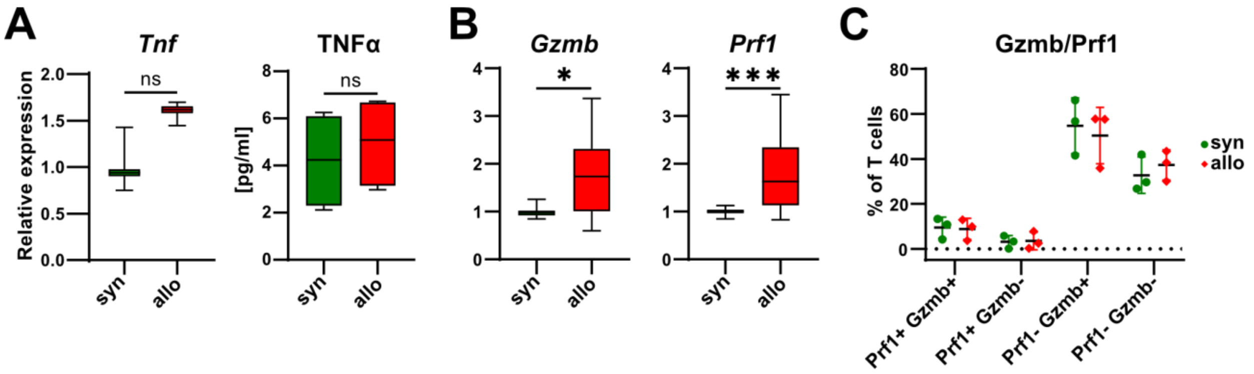

Supplementary Figure 1. Regulation of cytotoxic effector molecule expression under syngeneic vs. allogeneic IEL/IEC co-culture conditions.

SI organoids from allogeneic (Balb/c) or syngeneic (C57BL/6) donor mice were co-cultured with 2.5 x 10^5 C57BL/6 IELs enriched for $CD3^+$ and endpoint analyses were performed on d2 after start of the co-culture. On d2, cell-free supernatants were collected and cells from co-cultures were either harvested for RNA isolation (A-B) or single cell suspensions were used for flow cytometry (C).

(A) *Tnf* gene expression levels (left panel, n = 3) were determined by qPCR and were normalized so that the expression levels within the syngeneic condition equaled 1. Right panel depicts TNFα protein levels in cell-free supernatants detected by ELISA (n = 4). Graphs show median, minimum and maximum in box and whiskers plots of pooled data of indicated, independent experiments. For statistical analyses, two-tailed unpaired t test or t test with Welch's correction (left panel) was applied.

(B) Quantitative gene expression profiling for *Gzmb* (left, n = 12) and *Prf1* (right, n = 16) was performed by qPCR followed by normalization to the levels detected under the syngeneic co-culture condition. Graphs depict median, minimum and maximum in box and whiskers plots of pooled data, $*p \leq 0.05$, $*** p \leq 0.001$. Two-tailed unpaired t test or t test with Welch's correction was used for statistical assessment.

(C) Single cell suspensions were analyzed by flow cytometry for granzyme B (Gzmb) and perforin (Prf1) expression. Graph depicts the relative abundance (%) of Gzmb and Prf1 expressing cell subsets as indicated by excluding $TCR\beta^-$ $TCR\gamma\delta^-$ non-T cells through gating strategies. Data from n = 3 independent experiments were statistically assessed by two-way ANOVA and Šídák's multiple comparisons test.

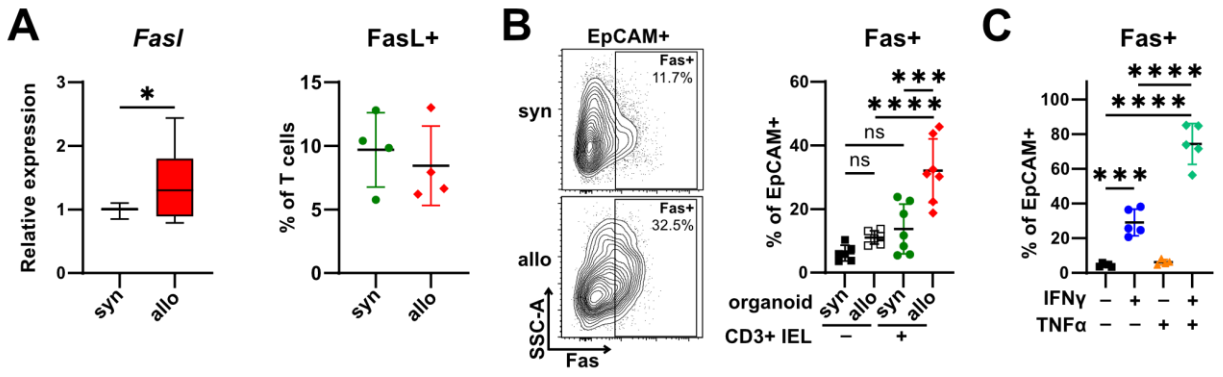

Supplementary Figure 2. IFNγ released from allo-activated IELs induces Fas expression on intestinal epithelial organoids.

(A) 2.5 x 10^5 C57BL/6 IELs enriched for $CD3^+$ were co-cultured with SI organoids from allogeneic (Balb/c) or syngeneic (C57BL/6) donor mice. 2d after start of the co-culture, cell-free supernatants were collected and cells from co-cultures were harvested. Left panel shows *Fasl* gene expression levels as determined by qPCR which were normalized so that the expression levels within the syngeneic condition equaled 1. Graph shows data from n = 10 independent experiments in a box and whiskers plot (median, minimum, maximum). *p ≤ 0.05 by t test with Welch's correction (left panel). Right panel depicts flow cytometric analysis of single cell suspensions of co-cultures on d2 for FasL+ cells within T cells (pre-gating by exclusion of $TCR\beta^-$ $TCR\gamma\delta^-$ non-T cells). Graph shows mean ± SD of n = 4 independent experiments statistically assessed by two-tailed unpaired t test.

(B) IEL/organoid co-cultures were generated and cultured under syngeneic and allogeneic conditions as described in (A). On d2, single cell suspensions of co-cultures were generated and analyzed for Fas expression within EpCAM+ IECs. Left panel shows representative data also illustrating the used gating strategy to determine Fas+ cell pools within all EpCAM+ cells in syngeneic vs. allogeneic condition. Right panel displays pooled data (mean ± SD) and statistical analysis of n = 7 independent experiments. *** p ≤ 0.001, **** p ≤ 0.0001 by one-way ANOVA and Šídák's multiple comparisons test.

(C) Organoids were cultured alone, i.e. without T cells. On d0, organoids were passaged and either left untreated or treated with recombinant murine TNFα (10 ng/ml), IFNγ (10 ng/ml) or both cytokines in the indicated concentration. On d2, organoid cultures were harvested and single cell suspensions were generated for flow cytometric analysis. Graph depicts pooled data from n = 5 independent experiments for the relative abundance (%) of Fas expressing cells in EpCAM+ IECs. *** p ≤ 0.001, **** p ≤ 0.0001 by one-way ANOVA and Šídák's multiple comparisons test.

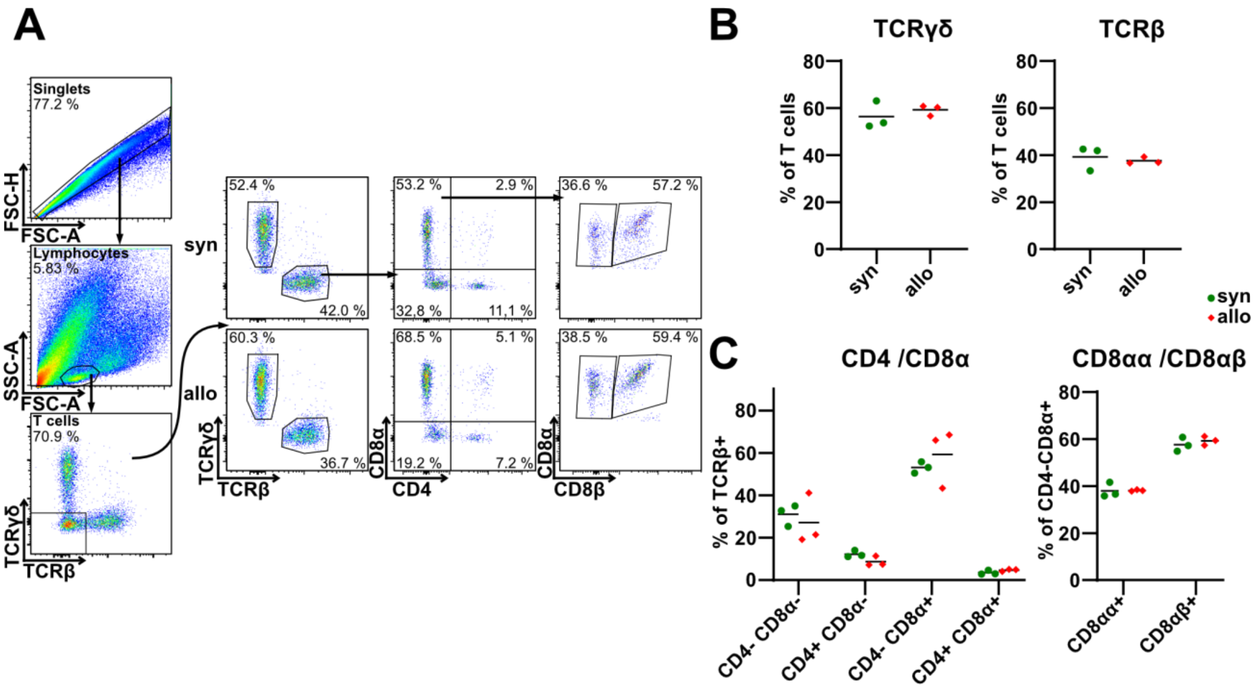

Supplementary Figure 3. Composition of IEL subpopulations after allogeneic vs. syngeneic co-culture with small intestinal organoids.

2.5 x 10^5 C57BL/6 IELs enriched for $CD3^+$ were co-cultured with SI organoids from allogeneic (Balb/c) or syngeneic (C57BL/6) donor mice and analyzed by flow cytometry 2d after start of the co-culture.

(A) Representative gating strategy showing exclusion of doublets, gating for lymphocytes and exclusion of non-T cells ($TCR\beta^-$ $TCR\gamma\delta^-$). Next, cells were gated for either $TCR\beta^+$ or $TCR\gamma\delta^+$. Within $TCR\beta^+$, $CD8\alpha^+$ vs. $CD4^+$ populations were assessed. Lastly, $CD8\alpha^+CD4^-$ cells were further examined for their composition of $CD8\alpha\alpha^+$ vs. $CD8\alpha\beta^+$ cells.

(B) Graphs depict the relative abundance (%) of $TCR\beta^+$ and $TCR\gamma\delta^+$ subsets within singlet lymphocytes after 2d of syngeneic or allogeneic co-culture.

(C) Relative abundance (%) of $TCR\beta^+$ (left) and $TCR\beta^+$ $CD8\alpha^+CD4^-$ (right) subsets within singlet lymphocytes after 2d of syngeneic or allogeneic co-culture.

Pooled flow cytometric results are from n = 3 independent experiments. Data in (B) were analyzed by two-tailed unpaired t test; data in (C) were statistically assessed by two-way ANOVA and Šídák's multiple comparisons test.
